# Pathogenic mechanism of abnormal expression of HDAC3 in ovulatory granulosa cells inducing oocyte maturation disorder and its application in IVM

**DOI:** 10.1016/j.jbc.2025.108287

**Published:** 2025-02-10

**Authors:** Huarong Wang, Han Cai, Meiling Zhang, Chuanhui Guo, Peike Wang, Na Deng, Haili Bao, Fanjing Meng, Qing Li, Shuiying Ma, Shuangbo Kong, Wenbo Deng, Hua Zhang, Guoliang Xia, Fengchao Wang, Chao Wang, Haibin Wang

**Affiliations:** 1 Department of Obstetrics and Gynecology, Guangdong Provincial Key Laboratory of Major Obstetric Diseases, Guangzhou, China; 2 Guangdong Provincial Clinical Research Center for Obstetrics and Gynecology, Guangzhou, China; 3 Guangdong-Hong Kong-Macao Greater Bay Area Higher Education Joint Laboratory of Maternal-Fetal Medicine, Guangzhou, China; 4 The Third Affiliated Hospital of Guangzhou Medical University, Guangzhou, China; 5 Fujian Provincial Key Laboratory of Reproductive Health Research, Department of Obstetrics and Gynecology, The First Affiliated Hospital of Xiamen University, School of Medicine, Xiamen University, Xiamen, China; 6 Center for Reproductive Medicine, International Peace Maternity and Child Health Hospital, Innovative Research Team of High-level Local Universities in Shanghai, School of Medicine, Shanghai Jiao Tong University, Shanghai, China; 7 Shanghai Key Laboratory for Assistant Reproduction and Reproductive Genetics, Center for Reproductive Medicine, Ren Ji Hospital, School of Medicine, Shanghai Jiao Tong University, Shanghai, China; 8 State Key Laboratory of Farm Animal Biotech Breeding, College of Biological Sciences, China Agricultural University, Beijing, China; 9 Center for Reproductive Medicine, The Second Hospital, Shandong University, Jinan, China; 10 Center for Reproductive Medicine, Shandong Provincial Hospital Affiliated to Shandong University, Jinan, China; 11 Key Laboratory of Ministry of Education for Conservation and Utilization of Special Biological Resources in the Western China, College of Life Science, Ningxia University, Yinchuan, Ningxia, China; 12 National Institute of Biological Sciences Beijing (NIBS), Beijing, China

**Keywords:** HDAC3, granulosa cell, LH, oocyte maturation, HDACi 4b, *in vitro* maturation

## Abstract

Female reproductive health is troubled by oocyte maturation disorder. In mammals, granulosa cells (GCs) mediate luteinizing hormone (LH) action on oocyte maturation and ovulation. However, the pathogenesis of disordered GCs in oocyte maturation arrest is rarely studied. Our previous study has showed that HDAC3 (histone deacetylase 3) in GCs was decreased by LH at physiological conditions. Here, we observed significantly elevated HDAC3 levels in GCs from patients with oocyte maturation disorder following LH treatment compared with those with normal oocyte maturation. To clarify whether abnormally high levels of HDAC3 in ovulatory GCs resulted in female infertility, a mice model of GC-conditional overexpression of *Hdac3* was constructed. The results showed that abnormally high levels of HDAC3 in ovulatory GCs inhibited LH induction on oocyte maturation and ovulation, resulting in female infertility. Further, in GCs with abnormal high levels of HDAC3, the upregulation of oocyte maturation–related genes induced by LH was attenuated by HDAC3 through a reduction in H3K14ac levels in the promoter regions, implying that the action of LH in GCs was largely negatively controlled by HDAC3. Applying HDAC3 inhibitors enhanced the expression of multiple genes associated with oocyte maturation in GCs from clinical patients, ultimately improving both the oocyte maturation rate and developmental quality, as demonstrated by a higher blastocyst development rate. The findings contribute to both enrich understanding upon the pathological mechanisms and supply optimal treatment strategies for patients with oocyte maturation disorder.

For female patients with reproductive defects, successful assistive reproductive technology (ART) outcomes depend largely on the availability of fully grown matured oocytes (1, 2). However, in the clinic, a portion of patients are infertile because of the inability to obtain mature oocytes after luteinizing hormone (LH) treatment (3, 4, 5, 6). For these patients, the general clinical strategy is to perform *in vitro* maturation (IVM) of the immature oocytes. Unfortunately, to date, the IVM outcome of these oocytes is generally poor (7). Therefore, it is important to uncover the pathogenesis of oocyte maturation disorder, which is helpful for the treatment of these patients.

Physiologically, the final stage of oocyte maturation requires close cooperation between oocytes and granulosa cells (GCs) within a fully grown follicle (8). Under the stimulation of follicle-stimulating hormone (FSH), GCs produce multiple maturation inhibitors such as C-type natriuretic peptide to maintain oocyte meiosis arrest (8, 9, 10, 11). Following the midcycle surge of LH induction, a transcriptome reprogramming in GCs takes place with activation of multiple signaling cascades for oocyte maturation properly (8, 12, 13). Consequently, the regulation of gene expression in GCs governs oocyte maturation (13, 14). Aberrant gene expression in GCs had been implicated in oocyte maturation arrest. For example, androgen induced C-type natriuretic peptide expression resulting in oocyte maturation arrest and ovulation failure in polycystic ovarian syndrome mice models (10). Notably, the pathological role of histone acetylation modification of GCs, one of the most critical epigenetic modifications in gene expression regulation, remains largely unexplored in oocyte maturation arrest (15, 16). As an important member of HDAC (histone deacetylase) family, the expression level of HDAC3 in GCs has been reported to be significantly downregulated by LH along with oocyte maturation (17). However, in clinical report and our studies in this article, the level of HDAC3 in GCs of patients who failed to achieve oocyte maturation and *in vitro* fertilization was significantly higher than that in *in vitro* fertilization successful patients (18). Therefore, this study was designed to explain whether abnormally elevated HDAC3 in GCs is the pathological factor resulting in female infertility.

In this study, using a mouse model with GC-specific overexpression of *Hdac3* (*Hdac3*^OE^), we demonstrated that abnormal HDAC3 expression in ovulatory GCs after the LH surge was a causative factor leading to female infertility. HDAC3 aberrant expression in ovulatory GCs hindered the expression of oocyte maturation–related genes under LH induction by reducing H3K14ac levels in their promoter regions. The HDAC3-specific inhibitor, HDACi4b, effectively suppressed HDAC3 activity in GCs, triggering the expression of multiple genes involved in oocyte maturation. This led to enhanced maturation and development of oocytes cocultured with HDACi4b-treated GCs from ART patients. These findings highlight the potential of the HDACi4b-GC coculture system as a viable approach to improve the IVM outcome of cultured oocytes, offering a promising strategy for clinical applications.

## Results

### Abnormal HDAC3 expression in GCs after LH induction impaired oocyte maturation and female fertility

**Figure 1:**
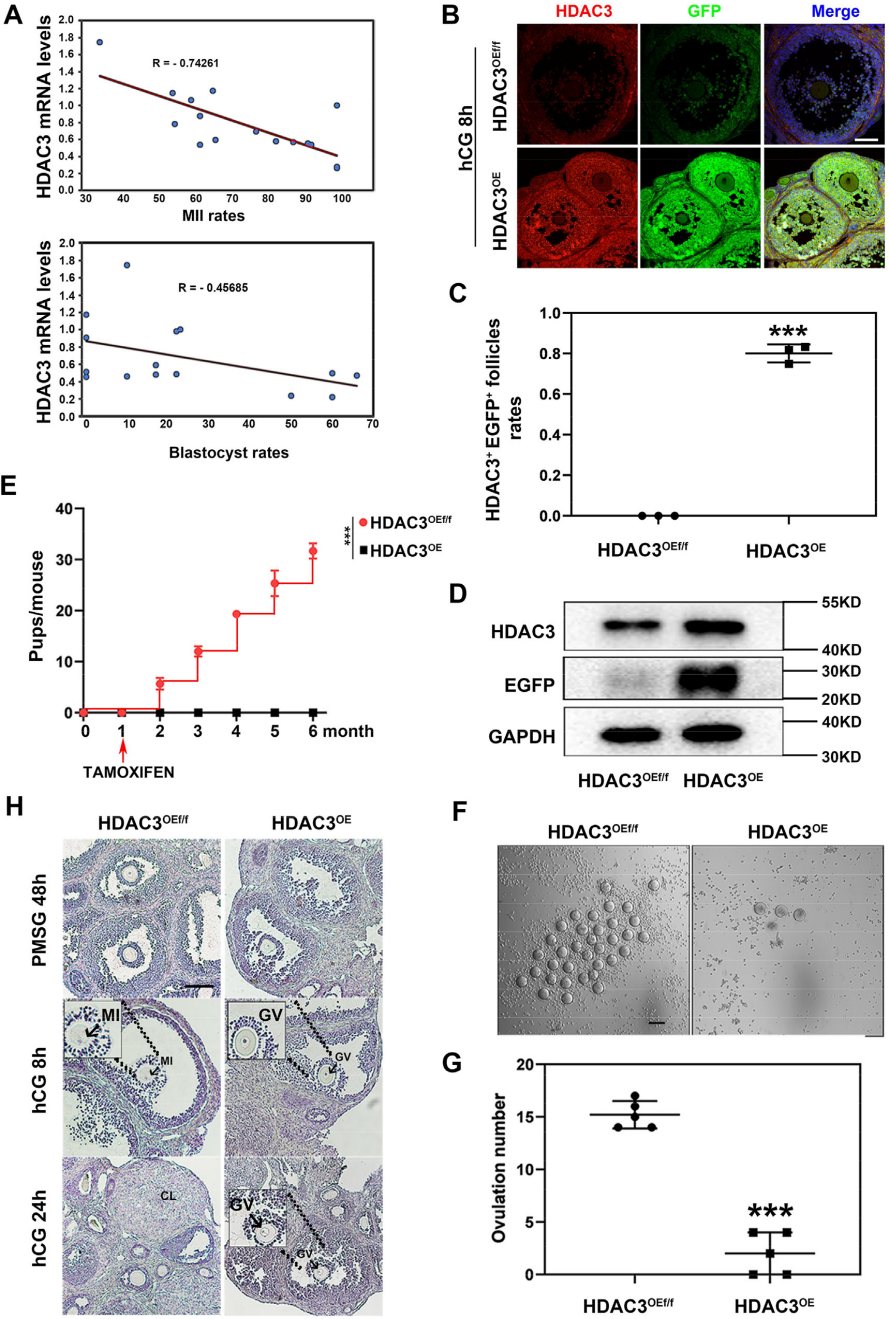
**Aberrant presence of HDAC3 in GCs of ovulatory follicles impaired oocyte maturation, ovulation, and female fertility.***A*, the mRNA levels of *HDAC3* in GCs derived from the patients correlated negatively with MII of oocyte rates and blastocyst rates. *B*, immunostaining analysis of EGFP and HDAC3 in ovulatory follicles 8 h after hCG treatment. HDAC3 was overexpressed successfully in *Hdac3*^OE^ GCs as compared with *Hdac3*^OEf/f^ GCs. *Green*: EGFP; *Red*: HDAC3; and *Blue*: DAPI. Scale bar represents 100 μm. *C*, follicle count statistics shown that approximately 80% of GCs in the ovulatory follicles in *Hdac3*^OE^ mice were HDAC3 and EGFP double positive. Graphs show the means ± SD of three independent experiments (n = 3). *p* Values were calculated using Student's *t* test (∗∗∗*p* < 0.001). *D*, immunoblotting analysis of EGFP and HDAC3 in *Hdac3*^OE^ GCs and *Hdac3*^OEf/f^ GCs 8 h after hCG induction. GAPDH was used as a loading control. *E*, fertility of *Hdac3*^OE^ and *Hdac3*^OEf/f^ female mice. Graphs show the means ± SD of three independent experiments (n = 3). *p* Values were calculated using Student's *t* test (∗∗∗*p* < 0.001). *F* and *G*, superovulation assays showed that the *Hdac3*^OE^ mice were unable to ovulate, as compared with the *Hdac3*^OEf/f^ mice. Scale bar represents 100 μm. Graphs show the means ± SD of three independent experiments (n = 3). *p* Values were calculated using Student's *t* test (∗∗∗*p* < 0.001). *H*, hematoxylin staining of ovaries from *Hdac3*^OEf/f^ and *Hdac3*^OE^ mice treated with PMSG for 48 h, hCG for 8 h, and hCG for 24 h, respectively. Scale bar represents 100 μm. CL, corpus luteum; DAPI, 4',6-diamidino-2-phenylindole; EGFP, enhanced GFP; GC, granulosa cell; GV, germinal vesicle; hCG, human chorionic gonadotropin; HDAC3, histone deacetylase 3; MII, metaphase II; PMSG, pregnant mare serum gonadotropin.

To explore whether elevated HDAC3 in ovulatory GCs serves as a pathogenic factor contributing to oocyte maturation disorder in the clinic, the mRNA levels of *HDAC3* in GCs obtained from these patients were examined. It showed that 36 h after LH induction, with the increase of *HDAC3* mRNA levels, the oocyte maturation efficiency and development ability of patients were significantly reduced (Fig. 1*A*). To further investigate whether the abnormally high expression of HDAC3 in ovulatory GCs is a key pathogenic factor for oocyte maturation disorder, a mouse model of *Hdac3* overexpression in ovulatory GCs was constructed.

Accordingly, *Rosa-LNL-Hdac3-P2A-Egfp* mice were generated with CRISPR technology (Fig. S1*A*) and crossed with the *Foxl2*-*Cre*ER^T2^ transgenic mice to get *Rosa-LNL-Hdac3-P2A-Egfp*; *Foxl2*-*Cre*ER^T2^ mice. Subsequently, these female crossbred mice, aged 18 days, were injected with tamoxifen (TAM) for three consecutive days to initiate the expression of the *Hdac3* transgene at puberty. Six days later, these mice were administered with pregnant mare serum gonadotropin (PMSG) and human chorionic gonadotropin (hCG) to induce a model of HDAC3 specifically expressed in ovulatory GCs after LH induction (*Hdac3*^OE^). As a control, the *Rosa-LNL-Hdac3-P2A-Egfp* transgenic mice without *Foxl2*-*Cre*ER^T2^ (*Hdac3*^OEf/f^) were used. To evaluate the efficiency of HDAC3 overexpression, we analyzed the expression of HDAC3 and enhanced GFP (EGFP) in ovulatory GCs from both control and *Hdac3*^OE^ mice 8 h after hCG treatment. As shown in Figure 1*B*, HDAC3 expression in the GCs of ovulatory follicles was nearly undetectable in control mice, with EGFP expression similarly absent. In contrast, GCs in more than 80% of ovulatory follicles in *Hdac3*^OE^ mice exhibited high levels of HDAC3 expression, accompanied by positive EGFP expression (Fig. 1*C*). The Western blotting results further confirmed the successful induction of HDAC3 overexpression in GCs of ovulatory follicles (Fig. 1*D*).

To interrogate the role of HDAC3 consecutive expression in ovulatory follicular GCs on female fertility, *Hdac3*^OE^ female mice and their littermate controls (*Hdac3*^OEf/f^) were respectively mated with wildtype fertile males. The breeding results showed that plug-positive *Hdac3*^OE^ female mice gave no birth, whereas the litter size in *Hdac3*^OEf/f^ mice was normal (Fig. 1*E*). Ovulatory assays further demonstrated that *Hdac3*^OE^ female mice were infertile because of the failure of ovulation (Fig. 1, *F* and *H*). Furthermore, we examined the development of follicles prior to ovulation to investigate the cause of ovulation disorder in *Hdac3*^OE^ mice. Briefly, LH administration induced oocyte maturation, cumulus cell expansion, ovulation, and corpus luteum (CL) formation as normal in *Hdac3*^OEf/f^ mice (Fig. 1*H*). However, in *Hdac3*^OE^ mice, no mature oocyte was produced as indicated by the complete lack of oocytes progressed to germinal vesicle (GV) breakdown (GVBD) stage, and no CL was available in the LH-primed ovaries (Fig. 1*H*). All these results in mice were consistent with the clinical observation that high levels of *HDAC3* in ovulatory GCs impaired LH-induced oocyte maturation in female patients (Fig. 1*A*). However, the IVM assays showed that oocytes isolated from *Hdac3*^OE^ mice underwent normal spontaneous maturation as those controls did, as were shown by the GVBD rates (Fig. S1*B*), indicating that the pathogenic mechanism of HDAC3 presence in ovulatory follicular GCs leading to female infertility is blocking LH-induced oocyte maturation and ovulation.

### HDAC3 in ovulatory GCs inhibited oocyte maturation by impeding LH-induced gene expression remodeling

**Figure 2:**
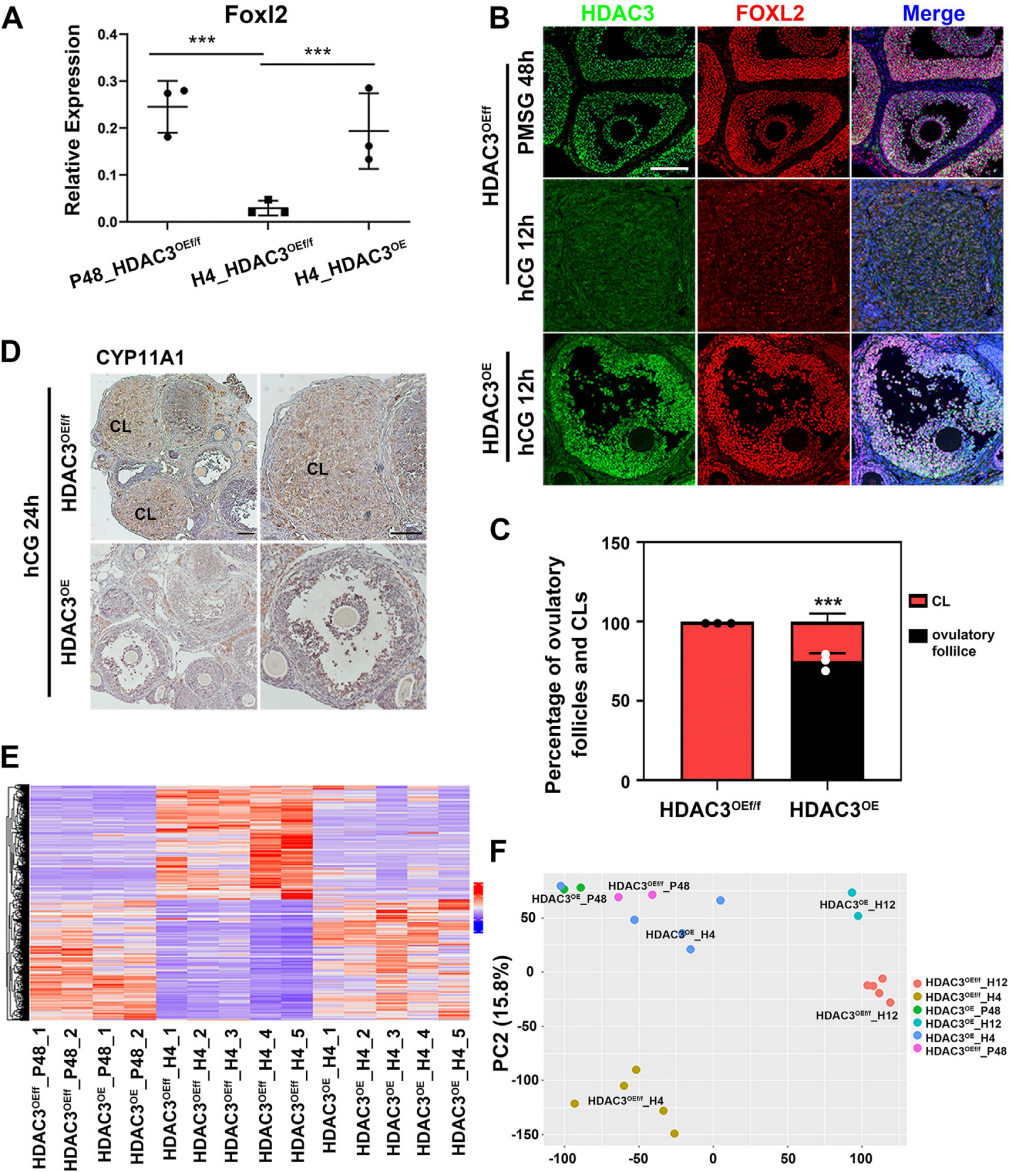
**HDAC3 blocked GC differentiation through impairing the reprogramming of GC transcriptome by LH.***A*, aberrantly elevated HDAC3 blocked the action of LH on downregulation of *Foxl2* mRNA levels. Graphs show the means ± SD of three independent experiments (n = 3). *p* Values were calculated using Student's *t* test (∗∗∗*p* < 0.001). *B*, the expression of FOXL2 protein in *Hdac3*^OE^ and *Hdac3*^OEf/f^ GCs was indicated by immunostaining assays. *Green*: HDAC3; *Red*: FOXL2; and *Blue*: DAPI. Scale bar represents 100 μm. *C*, ovulatory follicles and CLs statistics data shown that about 80% of ovulatory follicles in *Hdac3*^OE^ mice failed to ovulate to form CLs but still maintained follicular morphology with HDAC3 and FOXL2 double-positive GCs. Graphs show the means ± SD of three independent experiments (n = 3). *p* Values were calculated using Student's *t* test (∗∗∗*p* < 0.001). *D*, CYP11A1 immunohistochemistry stain showed that aberrantly elevated HDAC3 blocked the action of LH on upregulation of CYP11A1 expression. Scale bar represents 100 μm. *E* and *F*, overload of HDAC3 in ovulatory GCs blocked LH action on transcriptome reprogramming, as shown by and heatmap (*E*) and PCA plot (*F*). CL, corpus luteum; DAPI, 4',6-diamidino-2-phenylindole; GC, granulosa cell; HDAC3, histone deacetylase 3; LH, luteinizing hormone; PCA, principal component analysis.

The functional differentiation of GCs induced by LH is essential for oocyte maturation, ovulation, and CL formation (19, 20). To investigate whether abnormally high HDAC3 expression in ovulatory GCs is responsible for the dysfunction of GCs, FOXL2, an important functional marker of ovarian GCs, was used to detect the differentiation status of GCs (21). Quantitative real-time PCR analysis revealed that in *Hdac3*^OEf/f^ GCs, *Foxl2* mRNA levels were significantly reduced following hCG priming (Fig. 2*A*). Conversely, in *Hdac3*^OE^ mice, FOXL2 remained highly expressed in ovulatory follicle GCs after hCG treatment (Fig. 2, *A* and *B*). Immunostaining showed that 12 h after LH stimulation, FOXL2 and HDAC3 expression declined in control mice, facilitating CL formation. However, in *Hdac3*^OE^ mice, ∼80% of GCs retained high HDAC3 and FOXL2 expression, blocking ovulation and luteinization (Fig. 2, *B* and *C*). Immunostaining of CYP11A1 further confirmed impaired luteinization by 24 h post-hCG priming (Fig. 2*D*). GCs without luteinization in *Hdac3*^OE^ ovulatory follicles maintained their proliferative status, as indicated by immunofluorescence of Ki67 (Fig. S1*C*). All these results indicated that abnormally high HDAC3 expression in ovulatory GCs blocked LH-induced GC differentiation, resulting in failure of ovulation and CL formation.

Given that LH induces functional differentiation of GCs by altering gene expression patterns (13), therefore, RNA-Seq analysis was performed to investigate whether abnormal HDAC3 expression in ovulatory GCs blocked LH-induced gene expression (Fig. S2). Using *Hdac3*^OEf/f^ mice, we found that hCG treatment for 4 h significantly altered gene expression patterns in GCs compared with PMSG treatment for 48 h (Fig. S2*A*). For instance, *Areg*, *Ereg*, and *Ptx3* for oocyte maturation and cumulus expansion, *Pgr* for ovulation, and *Star* for steroidogenesis were all upregulated by hCG. While, genes such as *Nppc* for oocyte meiosis arrest, *Foxl2* and other genes for GC differentiation were all downregulated by hCG. Before the arrival of LH, the pattern of gene expression in *Hdac3*^OEf/f^ GCs was largely consistent with that in *Hdac3*^OE^ GCs (Fig. 2, *E* and *F*). However, the number of differentially expressed genes in *Hdac3*^OE^ GCs stimulated by LH was significantly lower than that in *Hdac3*^OEf/f^ GCs (Fig. S2*C* versus Fig. S2*A*), indicating that HDAC3 impaired LH action on GC transcriptome reprogramming. Therefore, following LH induction, significant differences in gene expression were observed between *Hdac3*^OE^ GCs and *Hdac3*^OEf/f^ GCs (Figs. 2, *E* and *F*; S2*B*).

According to this study, in *Hdac3*^OE^ GCs, 79.2% of genes that were to be upregulated by LH did not exhibit upregulated expression levels, including *Areg*, *Ereg*, *Btc*, *Pgr*, *Star* and other genes pivotal for oocyte maturation and ovulation (Fig. S2, *A*–*D*). In addition, the expression of LH-downregulated genes in *Hdac3*^OEf/f^ GCs remained at high levels in *Hdac3*^OE^ GCs, including *Nppc*, *Foxl2*, and other genes related to oocyte meiosis arrest (Fig. S2, *A*–*C* and *F*). Moreover, Gene Ontology (GO) analysis showed that HDAC3 not only inhibited LH-upregulated oocyte maturation and ovulation-related pathways but also maintained LH-suppressed GC-specific pathways (Fig. S2, *E* and *G*). All these results indicated that the aberrantly high expression of HDAC3 in ovulatory GCs impeded the functional differentiation of GCs by suppressing LH-induced gene expression alterations, leading to oocyte maturation disorder and failure of ovulation.

### Aberrantly high HDAC3 in ovulatory GCs inhibited LH-induced transcriptome remodeling through reducing H3K14ac and H3K27ac

**Figure 3:**
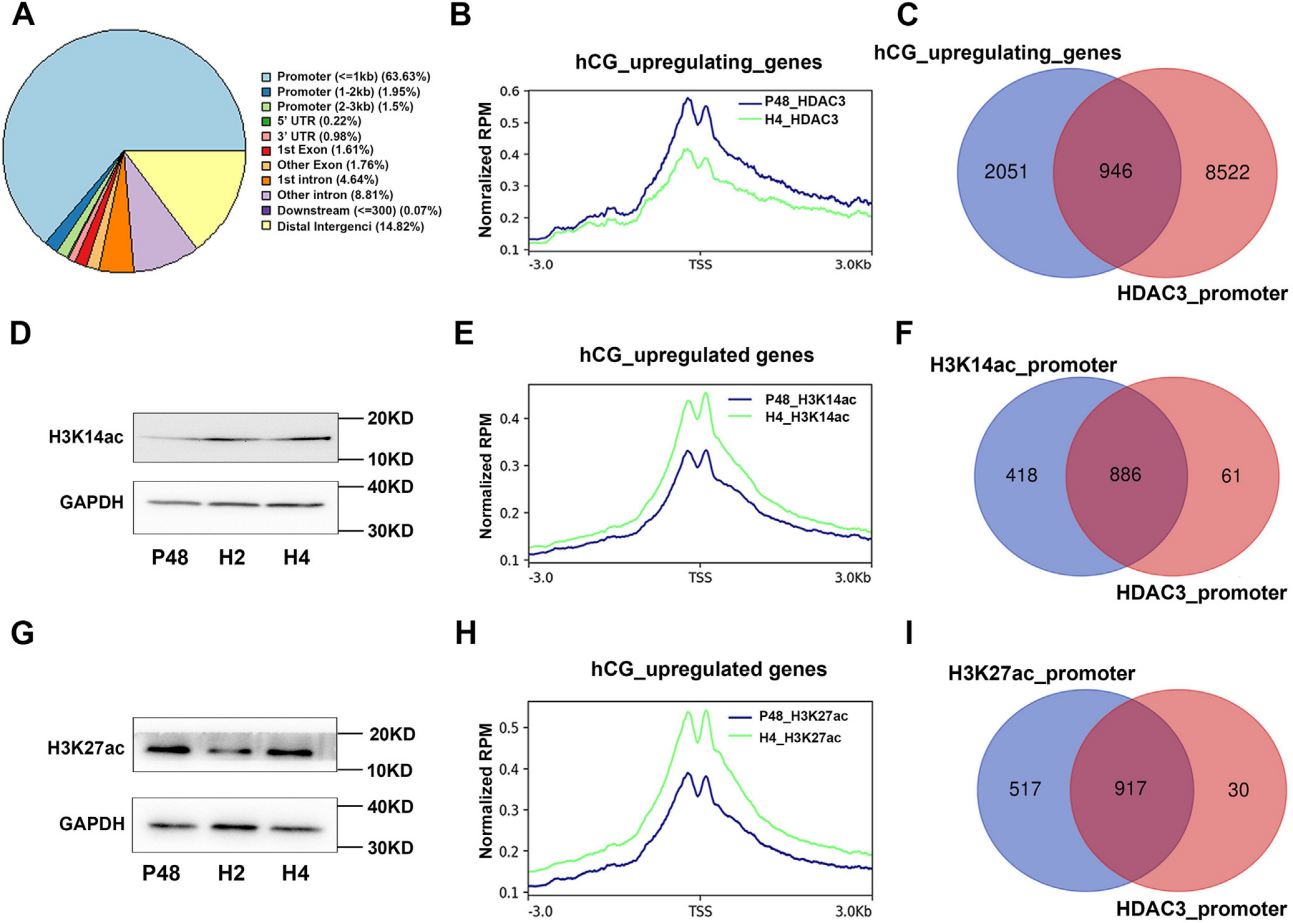
**HDAC3 and histone acetylation mediated the action of LH on target gene expression.***A*, genomic distribution of HDAC3 peaks in ovarian GCs before LH induction. *B*, HDAC3 CUT&Tag signal density at TSS of hCG-upregulated genes before and after LH induction. *C*, Venn diagram showing the overlap between genes upregulated by LH and genes with promoter bounded by HDAC3. *D*, immunoblotting analysis of H3K14ac in ovarian GCs before and after LH induction. GAPDH was used as loading controls. *E*, H3K14ac CUT&Tag signal density at TSS of hCG-upregulated genes before and after LH induction. *F*, Venn diagram showing the overlap between genes with H3K14 acetylation in the promoter of genes upregulated by LH and genes repressed directly by HDAC3. *G*, immunoblotting analysis of H3K27ac in ovarian GCs before and after LH induction. GAPDH was used as loading controls. *H*, H3K27ac CUT&Tag signal density at TSS of hCG-upregulated genes before and after LH induction. *I*, Venn diagram showing the overlap between genes with H3K27 acetylation in the promoter of genes upregulated by LH and genes repressed directly by HDAC3. hCG, human chorionic gonadotropin; HDAC3, histone deacetylase 3; GC, granulosa cell; LH, luteinizing hormone; TSS, transcriptional start site.

To explore the molecular mechanism by which HDAC3 inhibits LH-induced alterations in gene expression, we analyzed the variations in HDAC3 binding to chromatin in GCs before and after LH induction using cleavage under target & tagmentation (CUT&Tag) technology (22). In line with the LH-induced downregulation of HDAC3 expression levels, the binding peaks of HDAC3 on chromosomes exceeded 10,000 before LH stimulation (Fig. S3*A*). However, following LH stimulation, the binding peaks of HDAC3 on chromosomes nearly vanished altogether (Fig. S3, *A* and *B*). Before LH induction, the majority of HDAC3-binding sites in the genome were concentrated in the promoter regions (Fig. 3*A*), and the genes within the HDAC3-binding promoter regions included LH-upregulated genes, LH-downregulated genes, and LH-unresponsive genes (Figs. 3*C* and S3*C*). Following LH induction, the enrichment levels of HDAC3 were dramatically downregulated among all these genes’ promoter regions (Figs. 3, *B* and *C*; S3, *A*–*C*), indicating that HDAC3 probably exhibited distinct regulatory mechanisms for genes with varying LH response effects.

Given that HDAC3 typically functions by reducing histone acetylation modifications, we wondered whether histone acetylation modifications are involved in LH-induced remodeling of gene expression in GCs. Based on previous studies, H3K14 and H3K27 acetylation modifications play important roles in the regulation of gene expression in GCs (23). Therefore, H3K14 and H3K27 acetylation modifications were detected in control and *Hdac3*^OE^ mice ovarian GCs following LH induction in our study. Stimulation with LH significantly upregulated the acetylation level of histone H3K14 in ovarian GCs (Figs. 3*D* and S5*A*), which was similar to that of previous results (17). In contrast, the acetylation level of histone H3K27 remained largely unchanged under the same conditions (Figs. 3*G* and S5*B*). Correspondingly, following LH stimulation and the subsequent loss of HDAC3 expression, the peak number of H3K14ac was significantly increased (Fig. S3*D*), and the peak number of H3K27ac in the GCs was slightly reduced by LH (Fig. S3*G*). However, the enrichment levels of both H3K14ac and H3K27ac on the promoter regions in GCs were significantly increased (Fig. S3, *D*, *E*, *G* and *H*), suggesting their involvement in LH-induced gene expression changes in GCs. For LH-upregulated genes, most of the promoter regions of HDAC3-binding genes were acetylated by H3K14 and H3K27 after LH induction (Fig. 3, *C*, *F* and *I*). In the promoter regions of these genes, concurrent with the loss of HDAC3 binding (Fig. 3*B*), a substantial upregulation of H3K14 and H3K27 acetylation levels occurred (Fig. 3, *E* and *H*), indicating that H3K14ac and H3K27ac were involved in regulation of HDAC3 on LH-upregulated genes in GCs. However, for LH-downregulated genes, H3K14ac level was significantly upregulated, whereas H3K27ac level remained unchanged after LH induction (Fig. S3, *C*, *F* and *I*), suggesting that histone acetylation may not be involved in the regulation of HDAC3 on LH-downregulated gene expression.

**Figure 4:**
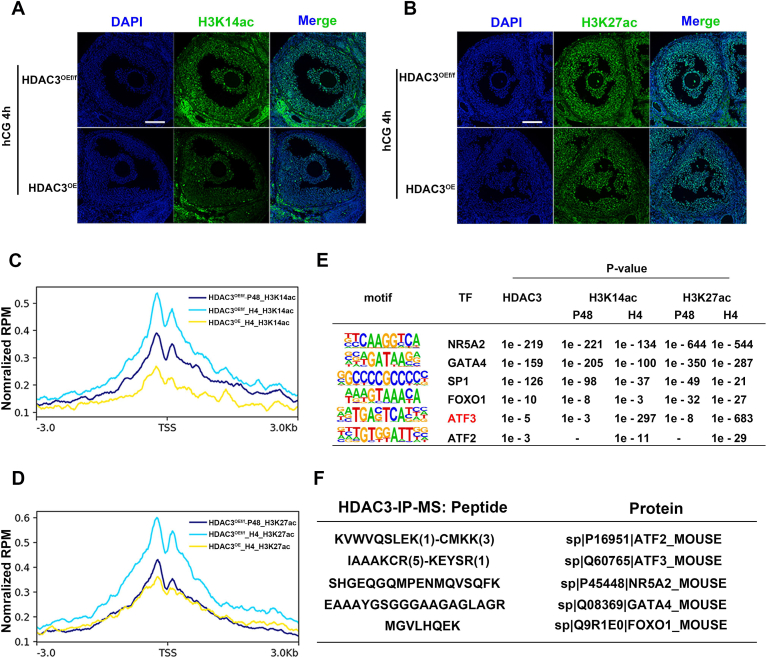
**Aberrantly upregulated HDAC3 in ovulatory GCs inhibited LH action through repressing H3K14ac and H3K27ac levels.***A*, immunostaining against H3K14ac showed that H3K14ac levels in *Hdac3*^OE^ GCs were significantly lower than *Hdac3*^OEf/f^ GCs 4 h after LH induction. *Green*: H3K14ac; *Blue*: DAPI. Scale bar represents 100 μm. *B*, immunostaining against H3K27ac showed that H3K27ac levels in *Hdac3*^OE^ GCs were significantly lower than *Hdac3*^OEf/f^ GCs 4 h after LH induction. *Green*: H3K27ac; *Blue*: DAPI. Scale bar represents 100 μm. *C*, H3K14ac CUT&Tag signal density at TSS of HDAC3 inhibited genes before and after LH induction in *Hdac3*^OE^ mice and the controls. *D*, H3K27ac CUT&Tag signal density at TSS of HDAC3 inhibited genes before and after LH induction in *Hdac3*^OE^ mice and the controls. *E*, de novo-enriched motifs at HDAC3-bound genomic regions. Statistics were determined by HOMER with one-sided hypergeometric *p* values for over-representation. *F*, proteins and peptides identified by HDAC3 immunoprecipitation (IP) coupled mass spectrum (MS). DAPI, 4',6-diamidino-2-phenylindole; GC, granulosa cell; HDAC3, histone deacetylase 3; LH, luteinizing hormone; TSS, transcriptional start site.

We further investigated the impact of abnormally high HDAC3 expression in GCs of ovulatory follicles on histone acetylation modification. About 4 h after hCG treatment, the protein levels and peak number of H3K14ac (Figs. 4*A*; S4, *A* and *C*) and H3K27ac (Figs. 4*B*; S4, *B* and *D*) were significantly reduced in *Hdac3*^OE^ mice. For LH-target genes repressed in *Hdac3*^OE^ GCs, the enrichment levels of H3K14ac and H3K27ac on their promoters were significantly lower than those in the control after LH–hCG induction (Fig. 4, *C* and *D*), indicating that abnormally high HDAC3 expression in ovulatory GCs repressed LH-target genes through downregulating histone acetylation.

As we know, HDAC3 recruited by different transcription factors exerts distinct regulatory mechanisms on target genes (24). For instance, HDAC3 recruited by ATF2 does not exhibit histone deacetylase activity to enhance gene expression, whereas HDAC3 recruited by ATF3 possesses histone deacetylase activity to suppress gene expression (24). In our previous reports, HDAC3-interacting proteins were analyzed using formaldehyde-linked coimmunoprecipitation and qualitatively assessed by mass spectrometry (17), and a large number of HDAC3-interacting proteins on chromosomes were identified. To determine which transcription factors mediated HDAC3 binding to chromosomes, we analyzed motifs of HDAC3-binding sites and HDAC3-associated proteins identified in our earlier study (Fig. 4, *E* and *F*). Many transcription factors, such as NR5A2, GATA4, SP1, and FOXO1, were reported to play important roles in LH-induced gene expression in GCs (Fig. 4, *E* and *F*) (17). In addition, ATF2 and ATF3 were identified. Notably, compared with ATF2 and other transcription factors, the ATF3 binding motif at histone acetylation sites was significantly enriched after LH-induced HDAC3 downregulation (Fig. 4*E*). This finding suggested that HDAC3 downregulation increased the acetylation levels of ATF3-binding sites. These results showed that the motifs of transcription factors such as ATF3 occupied the deacetylation active site of HDAC3, indicating that they may recruit HDAC3 with deacetylase activity to inhibit the expression of target genes, resulting in the failure of LH-induced gene expression.

### Inhibition of HDAC3 in GCs provided a new therapeutic approach to IVM by inducing the expression of multiple factors required for oocyte maturation

**Figure 5:**
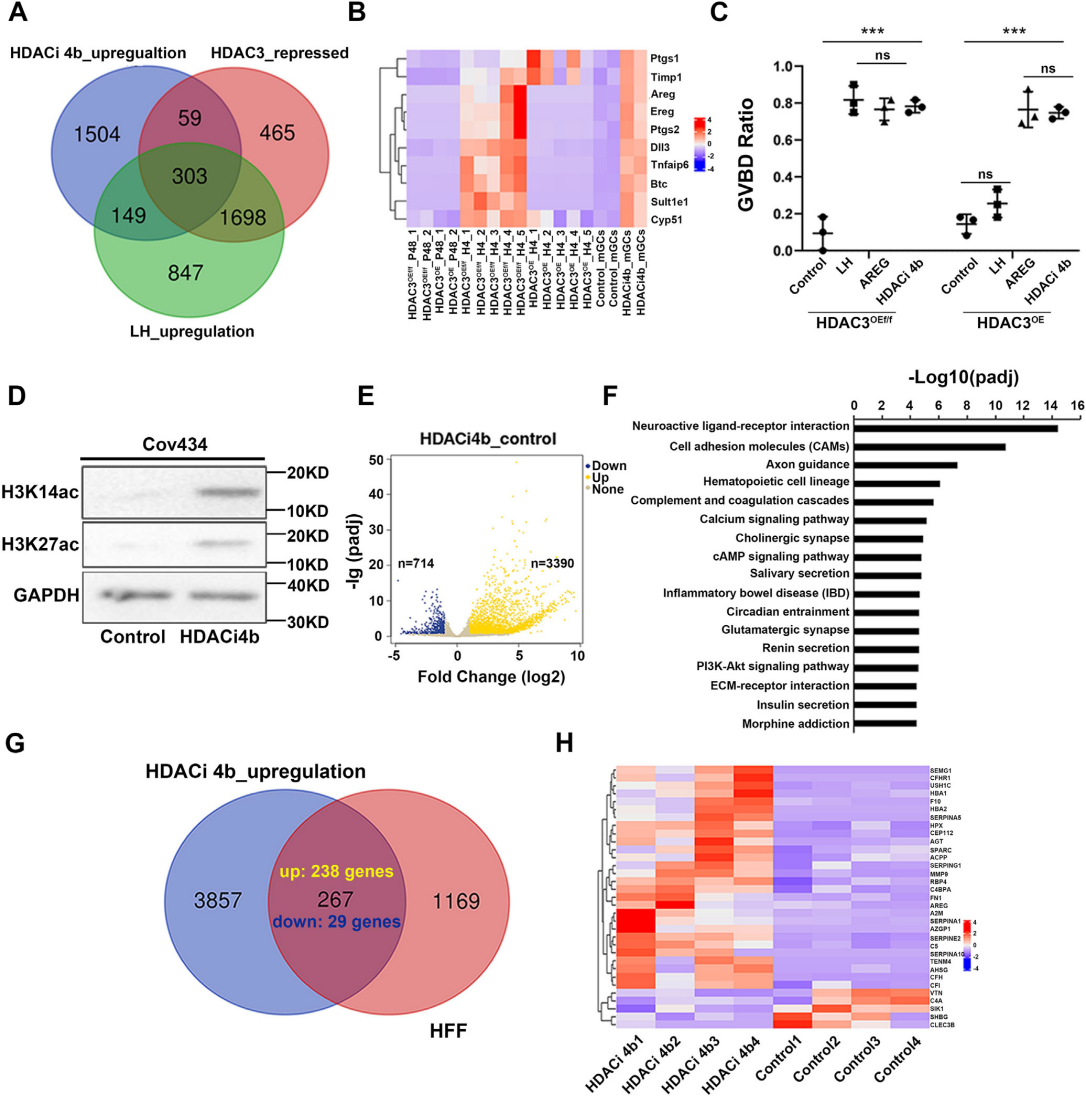
**HDAC3 inhibitor (HDACi)–induced GCs express multiple factors for oocyte maturation–independent LH induction.***A*, Venn diagram showing the overlap of HDACi 4b-inducible genes, LH-inducible genes, and HDAC3 inhibitory genes. *B*, heatmap listing several oocyte maturation factors belonging to HDACi 4b-inducible genes, LH-inducible genes, and HDAC3 inhibitory genes. *C*, GVBD ration analysis of oocytes in control and *Hdac3*^OE^ follicles treated with LH, AREG, and HDACi 4b. Graphs show the means ± SD of three independent experiments (n = 3). *p* Values were performed using ANOVA, ∗∗∗*p* < 0.001; ns = not significant. *D*, immunoblotting analysis showing that HDACi 4b increased the levels of H3K14ac and H3K27ac in human GC cell line (COV434). GAPDH was used as loading controls. *E*, Volcano plot showing that HDACi 4b induced dramatic changes of COV434 transcriptome with upregulation of 3390 genes. *F*, KEGG analysis of HDACi 4b-inducible genes in COV434. *G*, Venn diagram presenting overlap of HDACi 4b-inducible genes in COV434 and proteins identified in human ovulatory follicular fluids (HFFs). *H*, heatmap listing several high-confidence HFF proteins induced by HDACi 4b in COV434. AREG, amphiregulin; GC, granulosa cell; GVBD, germinal vesicle breakdown; HDAC3, histone deacetylase 3; KEGG, Kyoto Encyclopedia of Genes and Genomes; LH, luteinizing hormone.

In our previous work, we found that HDAC3 inhibitor (HDACi 4b) was able to stimulate GCs to promote oocyte maturation *in vitro* (17). Here, we further comprehensively analyzed the genes upregulated by LH and HDACi 4b in GCs as well as genes whose expression was significantly lower in *Hdac3*^OE^ GCs than in the control after LH stimulation (Fig. 5*A*). We found that oocyte maturation and ovulation-related genes such as EGF-like factors and *Ptgs1/2* were upregulated by LH and HDACi 4b in control GCs but not in *Hdac3*^OE^ GCs after LH induction (Fig. 5*B*). To verify whether aberrantly high HDAC3 induced oocyte maturation disorder by inhibiting the expression of oocyte maturation-related genes such as EGF-like factors, by using an *in vitro* follicle culture model, we found that in control follicles, oocyte maturation was induced by LH and amphiregulin (AREG) (Fig. 5*C*). However, in *Hdac3*^OE^ mouse follicles, LH was unable to induce oocyte maturation, but the addition of AREG could induce oocyte maturation as the control did (Fig. 5*C*), indicating that these oocyte maturation–related genes were indeed involved in HDAC3-induced oocyte maturation disorder. Moreover, HDACi 4b promoted oocyte maturation in both control and *Hdac3*^OE^ follicles through inducing the expression of multiple oocyte maturation–related genes in GCs *in vitro* (Fig. 5, *B* and *C*), suggesting that HDACi 4b had the potential to be used to improve clinical IVM system.

For human oocyte maturation, GCs secrete a variety of factors into human follicular fluid (HFF) in response to LH to constitute the microenvironment of oocyte maturation *in vivo*. To investigate whether HDACi 4b had similar effect in human GCs as in mice GCs, human GC line (COV434) was treated with HDACi 4b, which significantly increased the H3K14 and H3K27 acetylation levels of COV434 cells (Fig. 5*D*). RNA-Seq analysis showed that HDACi 4b induced dramatical changes of gene expression in COV434 with upregulation of 3390 genes (Fig. 5*E*). Kyoto Encyclopedia of Genes and Genomes analysis showed that pathways activated by HDACi 4b in COV434 were similar to that of LH in mice GCs (Fig. 5*F*). Furthermore, the transcriptome data of COV434 cells treated with HDACi 4b were deeply analyzed in combination with ovulatory HFF proteomics data (Fig. 5*G*). The expression levels of 267 genes encoding HFF proteins were changed significantly by HDACi 4b treatment, among which the expression levels of 238 genes were upregulated (Fig. 5*G*). The expression of part high-confidence HFF proteins was shown in Figure 5*H* indicating that HDAC3i was able to induce GCs to express multiple HFF factors required for oocyte maturation in human.

### Applying HDACi 4b *in vitro* improved maturation quality of oocytes retrieved from clinical patients

**Figure 6:**
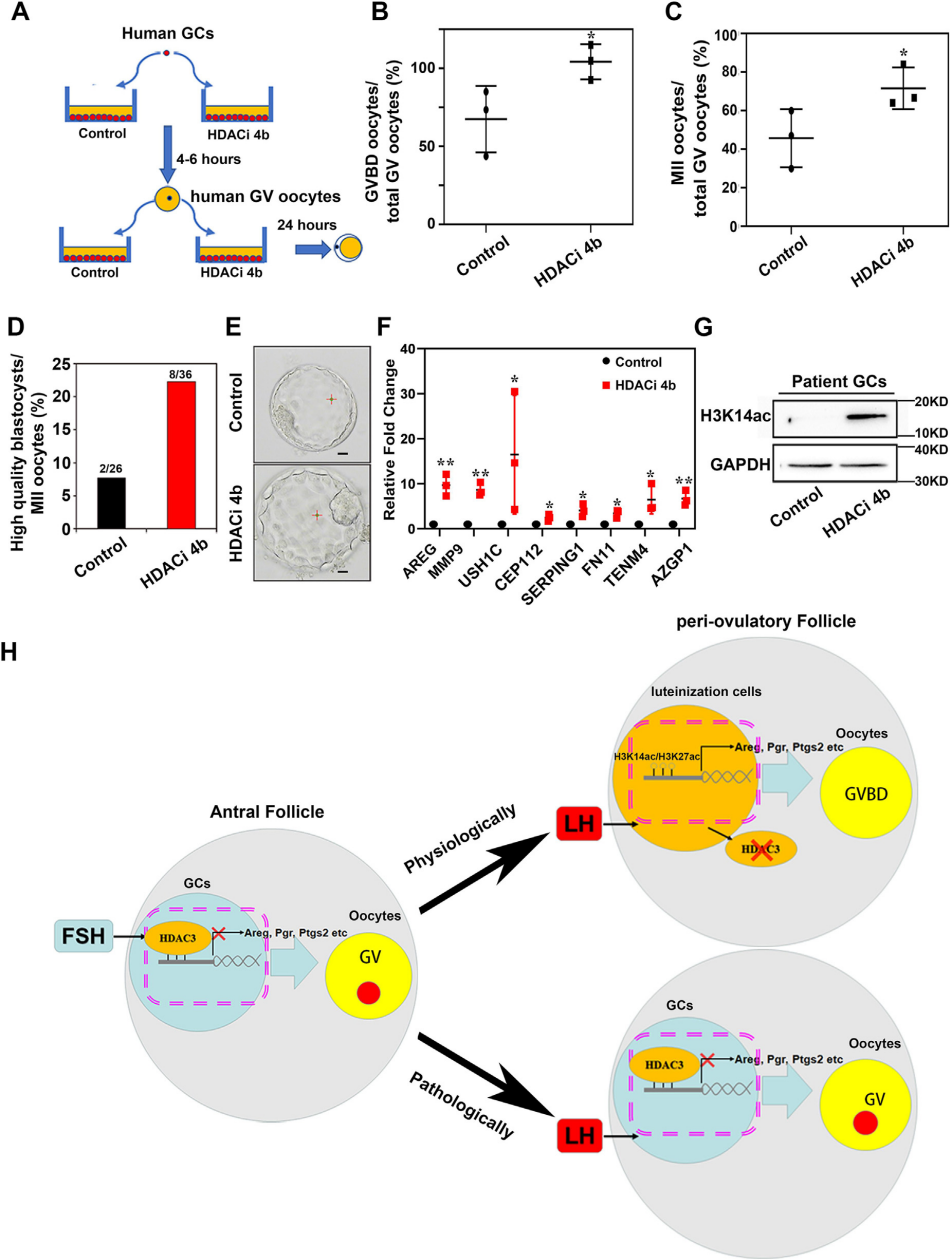
**Applying HDAC inhibitor (HDACi) 4b *in vitro* improved oocyte maturation quality in human.***A*, schematic overview of the improved IVM system with HDAC3i 4b. *B*, GVBD rates in HDACi 4b group were increased compared with those in the control. Scatter graphs show the means ± SD of three independent experiments (n = 3). *p* Values were calculated using Student's *t* test (∗*p* < 0.05). *C*, MII rates of GV-stage oocytes in HDACi 4b group were increased compared with those in the control. Scatter graphs show the means ± SD of three independent experiments (n = 3). *p* Values were calculated using Student's *t* test (∗*p* < 0.05). *D*, the ratios of high-quality blastocysts from MII-stage oocytes in HDACi 4b group were increased compared with those in the control. *E*, the quality of blastocysts obtained in HDACi 4b group (4AA) was improved compared with that in the control (4BB). Scale bar represents 100 μm. *F*, the mRNA levels of GC-secreted factors were increased significantly in HDACi 4b-treated GCs retrieved from clinical patients. Graphs show the means ± SD of three independent experiments (n = 3). *p* Values were calculated using Student's *t* test (∗*p* < 0.05, ∗∗*p* < 0.01). *G*, H3K14 acetylation levels in GCs retrieved from patients were increased by inhibiting action of HDACi 4b. GAPDH was used as loading controls. *H*, the proposed mechanism of HDAC3 in GCs mediated LH action on luteinization and oocyte maturation under physiological and pathological conditions. GV, germinal vesicle; GVBD, germinal vesicle breakdown; HDAC, histone deacetylase; IVM, *in vitro* maturation; MII, metaphase II.

Based on the application effects of HDACi 4b in mice GCs and in the human COV434 cell line as described previously, we wondered whether HDAC3 in ART patients’ ovarian GCs could be as a therapeutic target to improve IVM outcomes. Therefore, we developed a novel IVM system in which human GV-stage oocytes were cocultured with human GCs pretreated with HDACi 4b (Fig. 6*A*). To determine whether the IVM system with HDACi 4b-GCs was superior to the conventional clinical IVM system in improving oocyte maturation capacity, a total of 135 GV-stage cumulus–oocyte complexes (COCs) recovered from ART patients after LH induction were used. Briefly, GV-stage COCs were either cultured in clinical IVM medium as the control (25) or cultured on GC feeders from individual patients pretreated with HDACi 4b for 4 to 6 h. The results showed that the percentages of oocytes that progressed to the GVBD stage (50.6% ± 13.5% *versus* 83.4% ± 9.0%) and the metaphase II (MII) stage (45.7% ± 15.0% *versus* 71.6% ± 10.9%) were substantially higher in the HDACi 4b group than in the control (Fig. 6, *B* and *C*). More importantly, after oocytes were processed through intracytoplasmic sperm injection (ICSI) and embryo *in vitro* culture (IVC) procedures, many more high-quality blastocysts (blastocysts superior to grade 4) were obtained in the HDACi 4b group (2.9-fold) than in the control group according to the clinical standard (Gardner’s criteria) (26) (Fig. 6, *D* and *E*). Quantitative real-time PCR analysis showed that expression levels of many GC-secreted factors such as *AREG* were substantially increased along with the elevation of H3K14 acetylation in GCs retrieved from patients shortly after HDACi 4b treatment (Fig. 6, *F* and *G*), indicating that applying the HDACi 4b-GC feeder system resulted in better maturation quality and developmental capacity of immature oocytes from ART patients than the conventional clinical IVM system.

## Discussion

In this study, we found that aberrantly high expressed HDAC3 in ovulatory GCs after LH induction is an important pathogenic factor for female infertility (Fig. 6*H*). Physiologically, before LH arriving, the high expression of HDAC3 in GCs in response to FSH stimulation inhibits the expression of genes related to oocyte maturation and luteal differentiation, maintaining GC differentiation and oocyte maturation arrest. LH upregulated acetylation in the promoters of genes relative to oocyte maturation and luteal differentiation in GCs by downregulating HDAC3 expression, and subsequently activated the expression of LH target genes, thereby inducing oocyte maturation, ovulation, and luteinization. However, under pathological conditions, HDAC3 still presented at a high level after the arrival of LH in GCs, which inhibited the acetylation modification of the promoter regions of oocyte maturation–related genes, resulting in the inability to induce the expression of LH target genes and oocyte maturation disorder (Fig. 6*H*). Collectively, HDAC3 is essential for GC function before LH surge *in vivo*, and timely downregulation of HDAC3 in GCs is required for conducting LH signals to direct thorough GC differentiation and oocyte maturation. Our findings presented a representative example of HDAC3 on why and how epigenetic modification changes affect the outcome of pituitary signal–directed ovulation at either pathological conditions or physiological conditions. Based on the molecular mechanism of HDAC3 in GCs, we developed a novel IVM system with HDAC3i (HDACi 4b)-GC feeders. This IVM system significantly improved oocyte maturation microenvironment through inducing GC-expressed multiple HFF factors.

The follicle is the basic functional unit of the ovary. The development and maturation of follicles and oocytes require the coordination and interaction between GCs and oocytes, including endocrine, paracrine, and intercellular communication (27, 28, 29, 30, 31, 32). In female, FSH–LH secreted by the hypothalamic–pituitary gland are the key factors in inducing oocyte maturation (33). GCs in the follicle express both FSH and LH receptors. Before LH induction, GCs promote follicle development and inhibit oocyte maturation in response to FSH stimulation (34). After the arrival of LH, the gene expression and function of GCs switch from inhibiting oocyte maturation to promoting oocyte maturation and finally complete ovulation waiting for fertilization (34). Therefore, GCs are considered as the key factors supporting oocyte maturation *in vivo*, and abnormal GC function could lead to oocyte maturation failure, resulting in female infertility (34). Finding the pathogenic mechanism of GCs may improve the therapeutic effect of these patients by stimulating GCs *in vitro* to mimic the process of inducing oocyte maturation *in vivo*. In this study, we found that HDAC3 played a key role in LH-induced gene expression changes in GCs, and aberrantly high expressed HDAC3 completely blocked LH-induced gene expression changes by inhibiting the acetylation modification of LH target genes in the promoter regions. Based on the pathogenic mechanism of HDAC3 in GCs, we found that HDAC3i could induce the expression of a large number of oocyte maturation–related genes by promoting the acetylation modification of GCs derived from clinical ART patients, which significantly improved the therapeutic effect of IVM. In addition to HDACi 4b used in this study, other HDAC3is include RGFP966 and MS275 (35). RGFP966 is a specific inhibitor of HDAC3, whereas MS275 inhibits HDAC1, HDAC2, and HDAC3. Our *in vitro* data on oocyte maturation and development in mice ovulatory follicles revealed that the GVBD efficiency induced by RGFP966 and MS275 was comparable to that of HDACi 4b (Fig. S6*A*). However, the MII ratio for oocytes treated with RGFP966 and MS275 was slightly lower than that with HDACi 4b (Fig. S6*B*). Notably, *in vivo* administration of HDACi 4b in a Huntington’s disease mouse model showed minimal toxic side effects (36), supporting its potential as a clinical drug.

Although cumulative studies have been reported to improve the outcomes of the IVM of human oocytes, most of them rely on the effects of individual factors on oocyte maturation, such as EGF-like factors, melatonin, and growth hormone (25, 37). However, the poor IVM results imply that adding only a few factors into the IVM media may be insufficient to simulate the complexity of the *in vivo* maturation microenvironment because the oocyte maturation microenvironment *in vivo* is such a complicated niche that is composed of thousands of proteins/factors (14). For instances, the concentration of exogenously expressed AREG to the IVM media is as much as 20-fold higher than the natural content of the follicular fluid (3). Therefore, in this article, we found that targeting HDAC3 and employing GC feeders to produce natural flexible components *in vitro* could be an efficient measurement to optimize the present IVM strategy in the clinic.

## Experimental procedures

### Mice

C57BL/6J female mice were raised in colonies maintained by the investigators. Mice aged 21 to 23 days received an intraperitoneal (i.p.) injection of 5 IU of PMSG (Sansheng Pharmaceutical Co, Ltd) to stimulate follicle growth. About 46 to 48 h later, the mice received an i.p. injection of 5 IU of hCG (Sansheng Pharmaceutical Co, Ltd) to induce oocyte maturation and ovulation. *Foxl2*-*Cre*ER^T2^ mice were obtained as described before [19]. All these mice were housed under controlled temperature (22 °C) and light conditions (14 h light, 10 h darkness; lights on at 07:00 AM) and allowed free access to chow and water. Briefly, to obtain the *Hdac3*^OE^ mice, we crossed the *Rosa-LNL-Hdac3-P2A-Egfp* females with *Foxl2-Cre*ER^T2^ males. Then, female mice of *Rosa-LNL-Hdac3-P2A-Egfp*, *Foxl2-Cre*ER^T2^ were treated with TAM. Therefore, the offsprings with *Rosa-LNL-Hdac3-P2A-Egfp* but without *Foxl2-Cre*ER^T2^ were used as the controls. TAM was resuspended to a concentration of 100 mg/ml in 95% ethanol and further diluted with corn oil to a final concentration of 20 mg/ml. A single i.p. injection of TAM (20 mg/kg body weight) was administered respectively to 18-day-old female mice.

### *Rosa-LNL-Hdac3-P2A-Egfp* transgenic mice generation

Generation of *Rosa-LNL-Hdac3-P2A-Egfp* mice gRNAs was designed using the CRISPR guide-design web tool (https://crispr.dbcls.jp/). The selected gRNA sequence is ggcattctacacgttattgctgg. The targeting vector was synthesized by Genewiz, Inc. Mouse zygotes were obtained by mating of males with superovulated C57BL/6J females, were pronucleus injected with a mixture of Cas9 protein (80 ng/μl), sgRNA (40 ng/μl), and donor vector (10 ng/μl). Injected zygotes were transferred into pseudopregnant CD1 female mice. The correct recombinant mice were genotyped using PCR and sequencing.

### Breeding

We injected TAM to *Rosa-LNL-Hdac3-P2A-Egfp*, *Foxl2*-*Cre*ER^T2^ mice, and *Rosa-LNL-Hdac3-P2A-Egfp* mice at 30 days old, respectively, so as to generate the *Hdac3*^OE^ mice and the control *Hdac3*^OEf/f^ mice. About 10 days later, the *Hdac3*^OE^ mice and the *Hdac3*^OEf/f^ mice were crossed with wildtype male mice to detect their breeding ability, respectively.

### Immunofluorescence

Ovaries and follicles were fixed in cold 4% paraformaldehyde for 48 and 24 h, respectively. Then, the samples were dehydrated in ethanol and toluene, embedded in paraffin, and sectioned at 5 μm thickness. Ovaries were transferred to APES (3-aminopropyl-triethoxysilane)-treated microscope slides (ZLI-9001; Zhongshan Company) for immunofluorescence staining. In brief, sections were deparaffinized and rehydrated, and antigen retrieval was performed by microwaving for 15 min in 0.01% sodium citrate buffer (pH 6.0). For immunofluorescence analysis, sections were blocked with 10% normal donkey serum and incubated overnight at 4 °C with primary antibodies (Table S1) before incubation with Alexa Fluor 488- or 555-conjugated secondary antibodies (1:100 dilution; Invitrogen, Life Technologies) for 1 h at 37 °C and 4',6-diamidino-2-phenylindole (D9542; Sigma–Aldrich) as a nuclear counterstain. Samples were observed under a microscope.

### Immunohistochemistry

For immunohistochemistry, ovary sections were treated with 3% H_2_O_2_ in PBS for 20 min to quench endogenous peroxidase activity. Nonspecific binding was blocked with 5% bovine serum albumin in PBS. Sections were then incubated overnight at 4 °C with primary antibodies (Table S1), incubated for 20 min at room temperature with horseradish peroxidase–labeled goat anti-rabbit IgG (Zhongshan Company) and rinsed with PBS. The antibody complex was detected using DAB reagent according to the manufacturer’s instructions (Zhongshan Company). For histochemistry, sections were deparaffinized, rehydrated, and stained with hematoxylin.

### RNA preparation and analysis

RNA was isolated from GCs using TRIzol Reagent (Invitrogen, Life Technologies) according to the manufacturer's protocol. The quantity and quality of total RNA were assessed using a Nanodrop spectrophotometer (Thermo Scientific). Reverse transcription (Promega Reverse Transcription System) was performed using 1 μg of total RNA per sample. Quantitative PCR was conducted and analyzed on an ABI 7500 Sequence Detection System (Applied Biosystems) using a standard protocol. The primers used for test genes are listed in Table S2. For RNA-Seq analysis of ovarian GCs, total RNA extracted from ovarian GCs was analyzed by Huada Gene Technology Co, Ltd. Differential expression genes were normalized to fragments per kilobase of exon model per million mapped reads using the DEseq2 package in R with the criteria of fold change significantly greater than 2 and *p* < 0.05. The visualization of RNA-Seq data was done by ggplot2 package in R. GO enrichment analysis of differential expression genes was analyzed by using DAVID (the Database for Annotation, Visualization, and Integrated Discovery [https://david.ncifcrf.gov]). GO terms with corrected *p* < 0.05 were considered significantly enriched.

### Immunoblotting

Total protein was extracted in WIP (CellChip, BJ Biotechnology Co, Ltd) as recommended by the manufacturer. In brief, total protein from each sample was separated by 10% SDS-PAGE and then electrophoretically transferred onto polyvinylidene fluoride membranes (IPVH00010; Millipore). The membranes were then incubated for 1 h at room temperature with 5% bovine serum albumin, followed by an overnight incubation at 4 °C with antibodies of anti-HDAC3/H3K14ac/H3K27ac/EGFP/GAPDH (Table S1). After being washed in Tris-buffered saline with Tween-20, the membranes were incubated with secondary antibody (1:5000 dilution, ZB-2301 and ZB-2305; ZSGB-BIO) in Tris-buffered saline with Tween-20, followed by detection with the SuperSignal chemiluminescent detection system (34080; Thermo Scientific). GAPDH was used as an internal control.

### CUT&Tag

The CUT&Tag procedure was adapted and conducted following the NovoNGS CUT&Tag 4.0 High-Sensitivity Kit (Novoprotein; N259-YH01). Briefly, as many as 1 × 10^5^ cells were harvested and washed twice in 500 μl wash buffer at room temperature. Concanavalin A-coated magnetic beads were prepared as the kit manual described, and 10 μl of activated beads were added per sample and incubated at ROOM temperature for 10 min. According to the kit instructions, cells were sequentially incubated with ConA beads, primary antibody (anti-HDAC3 antibody, anti-H3K14ac antibody, and anti-H3K27ac antibody), secondary antibody, and hyperactive PG-TN5/PA-TN5 transposon and then fragmented. The fragmented DNA was extracted from the samples and amplified by PCR. CUT&Tag libraries were constructed and sequenced on an Illumina NovaSeq platform, 150-bp paired-end reads were generated. Fastp v 0.20.0 was used to remove adapter and low-quality reads. Align paired-end reads using Bowtie2, version 2.3.4.3 with options: end-to-end sensitive. Duplicated reads are removed using Picard, v2.18.17 with this parameter: REMOVE_DUPLICATES = true. Peak calling uses SEACR v1.3 with threshold: 0.01. Scatterplots, correlation plots, and heatmaps are displayed using deepTools, v2.27.1. Annotation of peaks is performed using an R package ChIPseeker, v1.12.1. MEME-ChIP, v 5.0.5 was used to search for the binding site. Peaks with *M* value >0.2 and *p* value <0.05 are defined as specific peaks.

### Cell line culture

COV434 cell line was purchased from Shanghai Yuanye Bio-Technology Co, Ltd and cultured in RPMI1640 medium (catalog no.: 11875085; Life Technologies) with 10% fetal bovine serum (Life Technologies).

### Follicle culture

Follicles were isolated from the ovaries of PMSG-primed mice and cultured in M199 medium with insulin–transferrin–sodium selenite media supplement. To investigate whether HDAC3i (HDACi 4b) (sc207902) could overcome HDAC3 inhibitory on oocyte maturation *in vitro*, both control follicles and *Hdac3*^OE^ follicles were cultured for 14 h with or without 10 μM HDACi 4b under LH induction. AREG was added to these follicles as a positive control. Oocyte maturation ratios in all these groups were examined at the end of culture.

### Human oocyte IVM, ICSI, and embryo IVC

Before these assays were started, informed consent was obtained from all participants. All the participating patients underwent ART based on standard ovarian stimulation protocols. The participants included the following types of patients: healthy female patients with infertile husbands, infertile female patients with tubal blockage, or infertile female patients with ovarian diseases. Similar to the methods performed with mice in this study, all the GV-stage COCs retrieved from gonadotropin-primed patients were divided into two groups and cocultured with human ovarian GC feeders in IVM medium containing 10% human serum albumin with or without 10 μM HDACi 4b. Oocytes that developed to the MII stage were used for ICSI. The zygotes and the early development embryos were cultured with G1 plus medium and G2 plus medium, respectively, before examination.

### Study approval

The experiments were performed in accordance with the principles and guidelines for the use of laboratory animals of Xiamen University and approved by the Institutional Animal Care and Use Committee of Xiamen University. All experiments using human samples were conducted in accordance with Renji Hospital and Shandong Province University. The protocols for handling IVM of human GV-staged oocytes, ICSI, and early embryo IVC were approved by the Institutional Ethics Committee of the Renji Hospital Affiliated to Shanghai Jiaotong University and Center for Reproductive Medicine Affiliated to Shandong Province University. Human studies reported in our article abide by the Declaration of Helsinki principles.

### Statistical analysis

The data for RT–PCR assays, follicle and oocyte counting experiments, and breeding tests are presented as the mean ± SD, and each experiment was performed in triplicate. Data were analyzed using *t* test or ANOVA. Values of *p* < 0.05 were considered statistically significant.

## Data availability

All data generated or analyzed during this study are available upon request.

## Supporting information

This article contains supporting information.

## Conflict of interest

The authors declare that they have no conflicts of interest with the contents of this article.

## Supporting information

Supporting Information-1.9

Rawdata-1.6

Cut & tag data

RNA-seq data
