# Supplementary material for: Pathogenic mechanism of abnormal expression of HDAC3 in ovulatory granulosa cells inducing oocyte maturation disorder and its application in IVM
Source: J Biol Chem. 2025 Feb 10;301(3):108287. doi: 10.1016/j.jbc.2025.108287 (PMC11923827; doi:10.1016/j.jbc.2025.108287)
Supplement: Supporting Information-1.9 [file mmc1.docx]

**Pathogenic mechanism of abnormal expression of HDAC3 in ovulatory granulosa cells inducing oocyte maturation disorder and its application in IVM**

Huarong Wang^#^, Han Cai^#^, Meiling Zhang^#^, Chuanhui Guo, Peike Wang, Na Deng, Haili Bao, Fanjing Meng, Qing Li, Shuiying Ma, Shuangbo Kong, Wenbo Deng, Hua Zhang, Guoliang Xia, Fengchao Wang, Chao Wang*, Haibin Wang*

**^#^** The authors contributed equally to this article.

* Authors for correspondence (wangcam@126.com; haibin.wang@vip.163.com)

This PDF file includes:
Figures S1 to S6 and Tables S1 to S2
Figures Legends for S1 to S6 and Tables S1 to S2


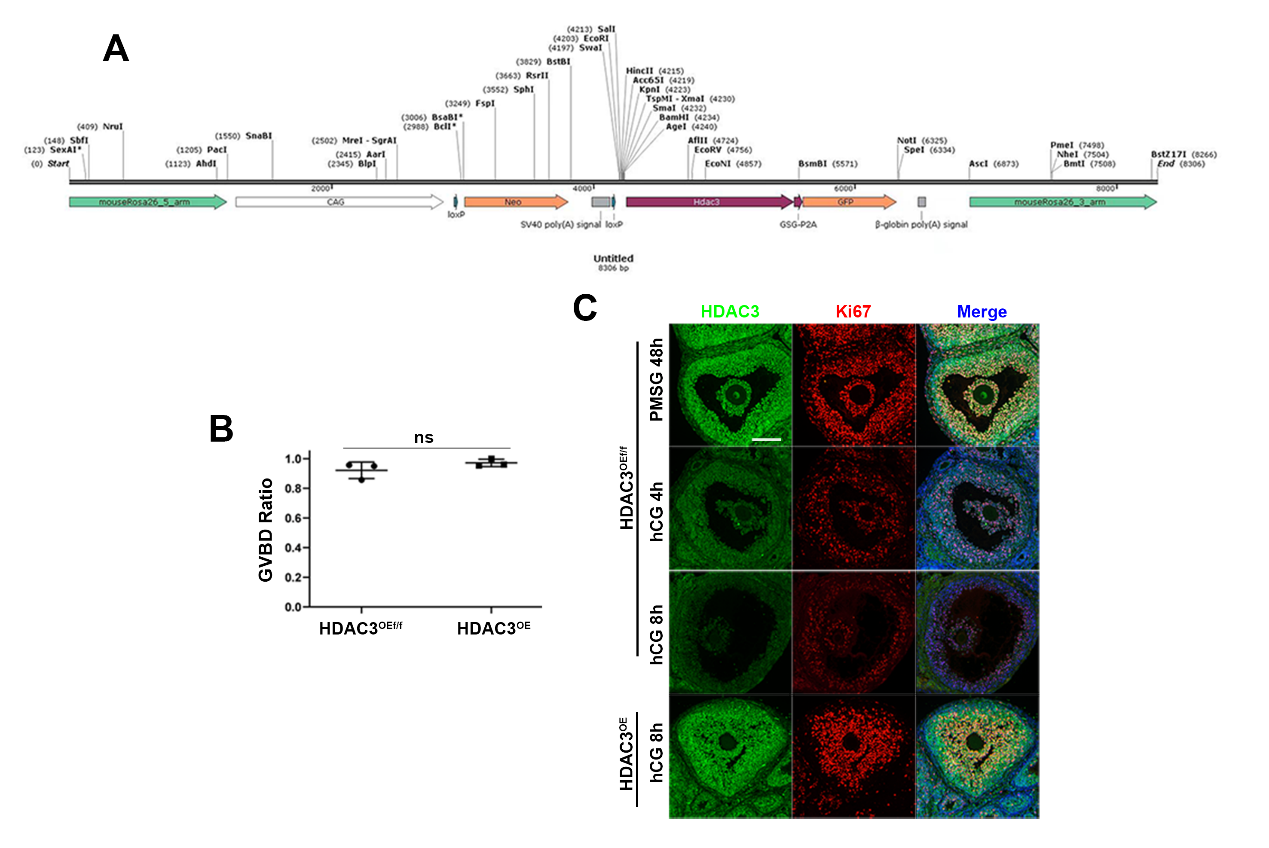


**Fig S1. HDAC3 inhibited LH action on GCs.** (**A**) Vector diagram required for generating *Hdac3* overexpression transgene mice. (**B**) IVM assay of oocytes from *Hdac3*^OEf/f^ and *Hdac3*^OE^ mice treated with PMSG for 48 hours. Graphs show the means ± SD of three independent experiments (n=3). *P*-values were calculated using Student´s *t*-test (ns = not significant). (**C**) Immunostaining against HDAC3 and Ki67 of ovaries from *Hdac3*^OEf/f^ and *Hdac3*^OE^ mice treated with PMSG for 48 hours, hCG for 4 hours and hCG for 8 hours, respectively. Green: HDAC3; Red: Ki67; Blue: DAPI. Scale bar: 100 μm.

**
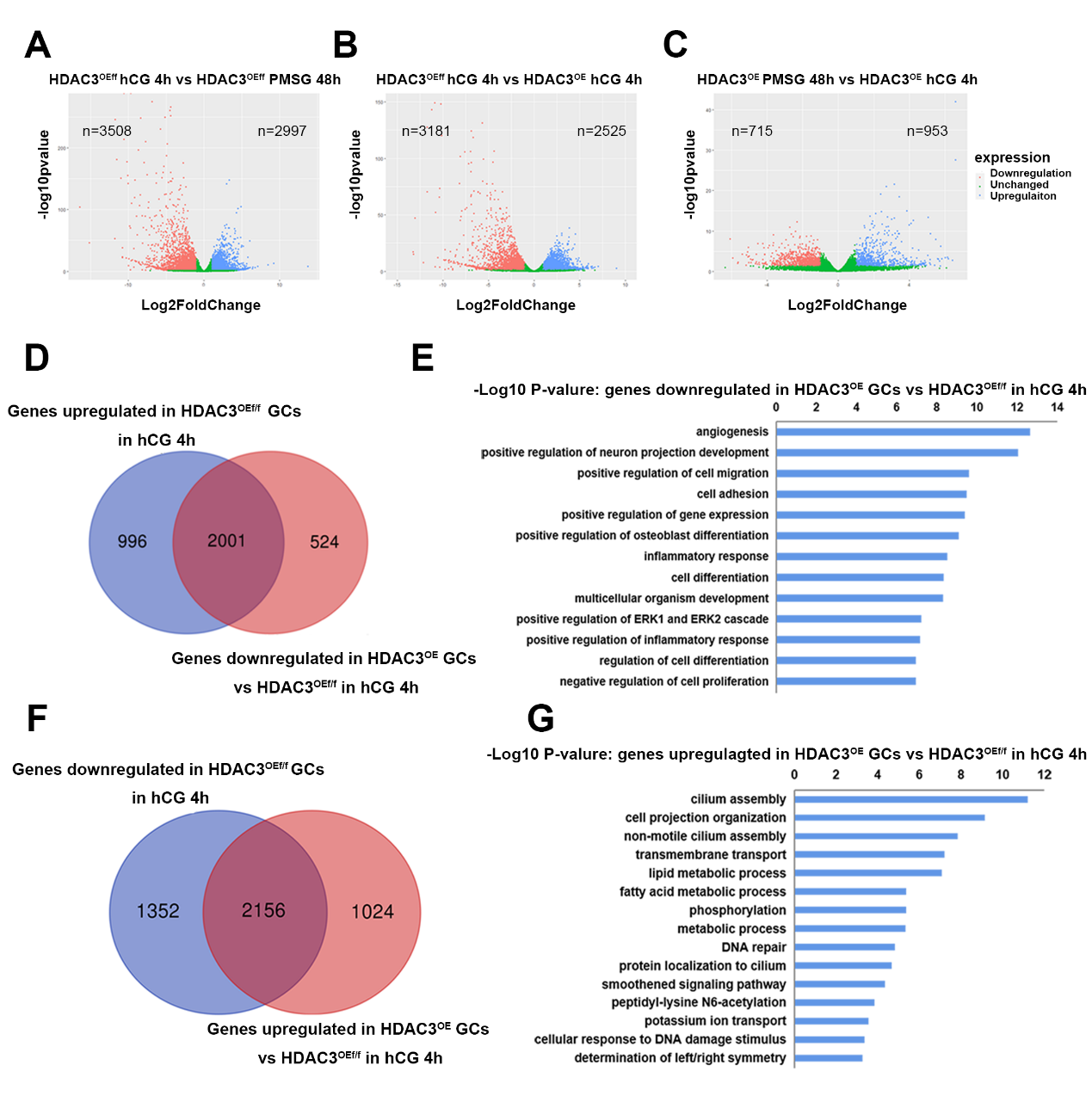
**

**Fig S2. HDAC3 repressed LH action on GC transcriptome reprogramming in mice.** (**A**) Volcano plot showing the different expression genes in *Hdac3*^OEf/f^ ovarian GCs before and after LH induction. (**B**) Volcano plot showing the different expression genes between *Hdac3*^OEf/f^ ovarian GCs and *Hdac3*^OE^ ovarian GCs 4 hours after hCG treatment. (**C**) Volcano plot showing the different expression genes in *Hdac3*^OE^ ovarian GCs before and after LH induction. (**D**) Venn diagram representing the genes overlapped by genes upregulated by LH in *Hdac3*^OEf/f^ ovarian GCs and genes downregulated in *Hdac3*^OE^ ovarian GCs versus *Hdac3*^OEf/f^ ovarian GCs. (**E**) GO analysis of genes downregulated in *Hdac3*^OE^ ovarian GCs versus *Hdac3*^OEf/f^ ovarian GCs. (**F**) Venn diagram representing the genes overlapped by genes downregulated by LH in *Hdac3*^OEf/f^ ovarian GCs and genes upregulated in *Hdac3*^OE^ ovarian GCs versus *Hdac3*^OEf/f^ ovarian GCs. (**G**) GO analysis of genes upregulated in *Hdac3*^OE^ ovarian GCs versus *Hdac3*^OEf/f^ ovarian GCs.


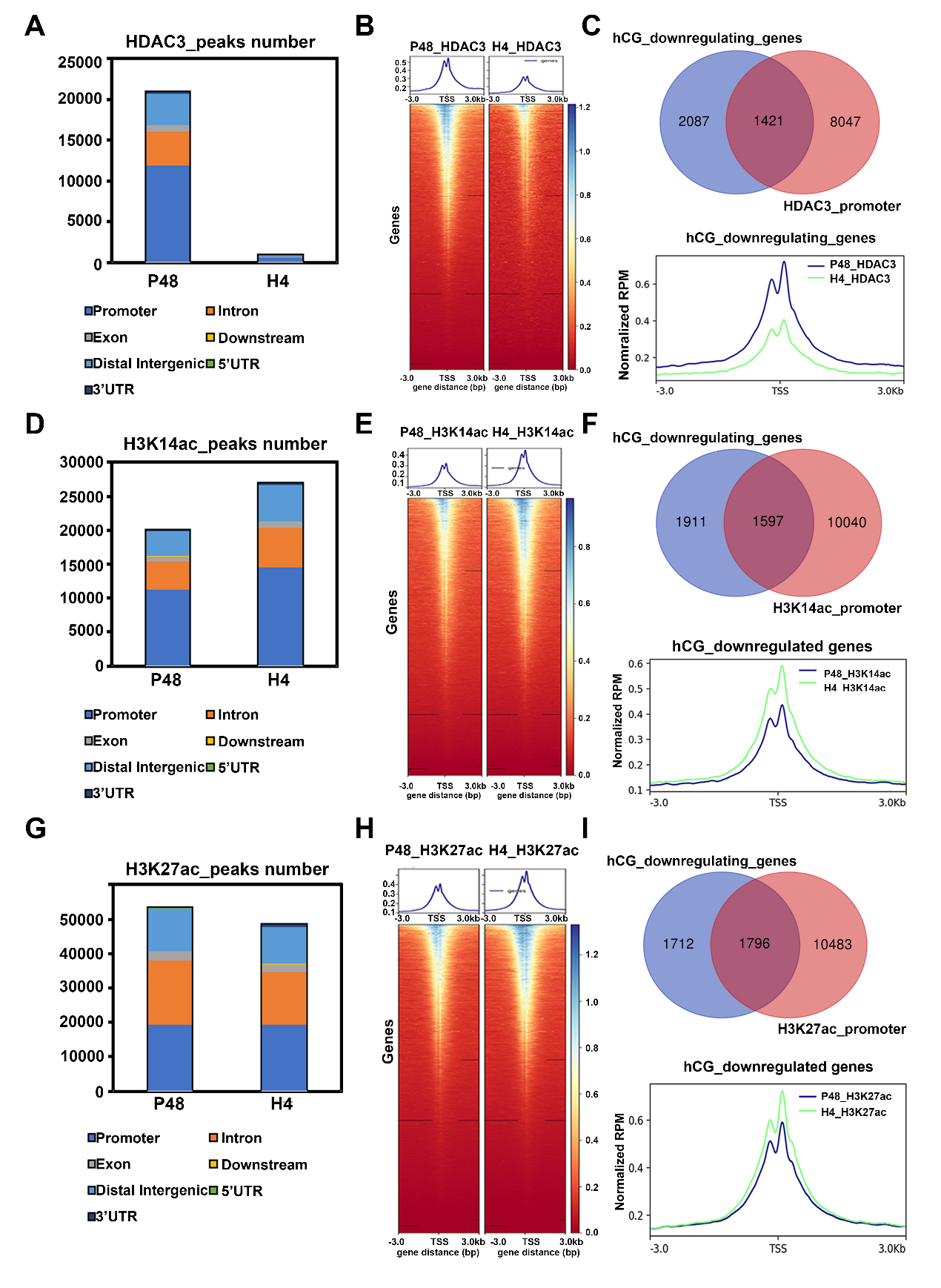


**Fig S3. HDAC3 and histone acetylation mediated LH action on GC gene expression.** (**A**) Diagram representing the peaks number of HDAC3 on the chromatins in *Hdac3*^OEf/f^ GCs before and 4 hours after hCG induction. (**B**) Coverage profiles for HDAC3 enrichment on TSS before and after LH induction. Heatmap of peaks with decreased HDAC3 following LH induction centered at TSS. TSS, transcriptional start site. (**C**) Venn analysis and Cut & Tag signal density showing that LH significantly decreased enrichment levels of HDAC3 binding on the promoters of LH-downregulating genes. TSS, transcriptional start site. (**D**) Diagram representing the peaks number of H3K14ac on the chromatins in *Hdac3*^OEf/f^ GCs before and 4 hours after hCG induction. (**E**) Coverage profiles for H3K14ac enrichment on TSS before and after LH induction. Heatmap of peaks with increased H3K14ac following LH induction centered at TSS. TSS, transcriptional start site. (**F**) Venn analysis and Cut & Tag signal density showing that LH significantly increased enrichment levels of H3K14ac binding on the promoters of LH-downregulating genes. TSS, transcriptional start site. (**G**) Diagram representing the peaks number of H3K27ac on the chromatins in *Hdac3*^OEf/f^ GCs before and 4 hours after hCG induction. (**H**) Coverage profiles for H3K27ac enrichment on TSS before and after LH induction. Heatmap of peaks with increased H3K27ac following LH induction centered at TSS. TSS, transcriptional start site. (**I**) Venn analysis and Cut & Tag signal density showing that LH had no significantly effect on the enrichment levels of H3K27ac binding on the promoters of LH-downregulating genes. TSS, transcriptional start site.


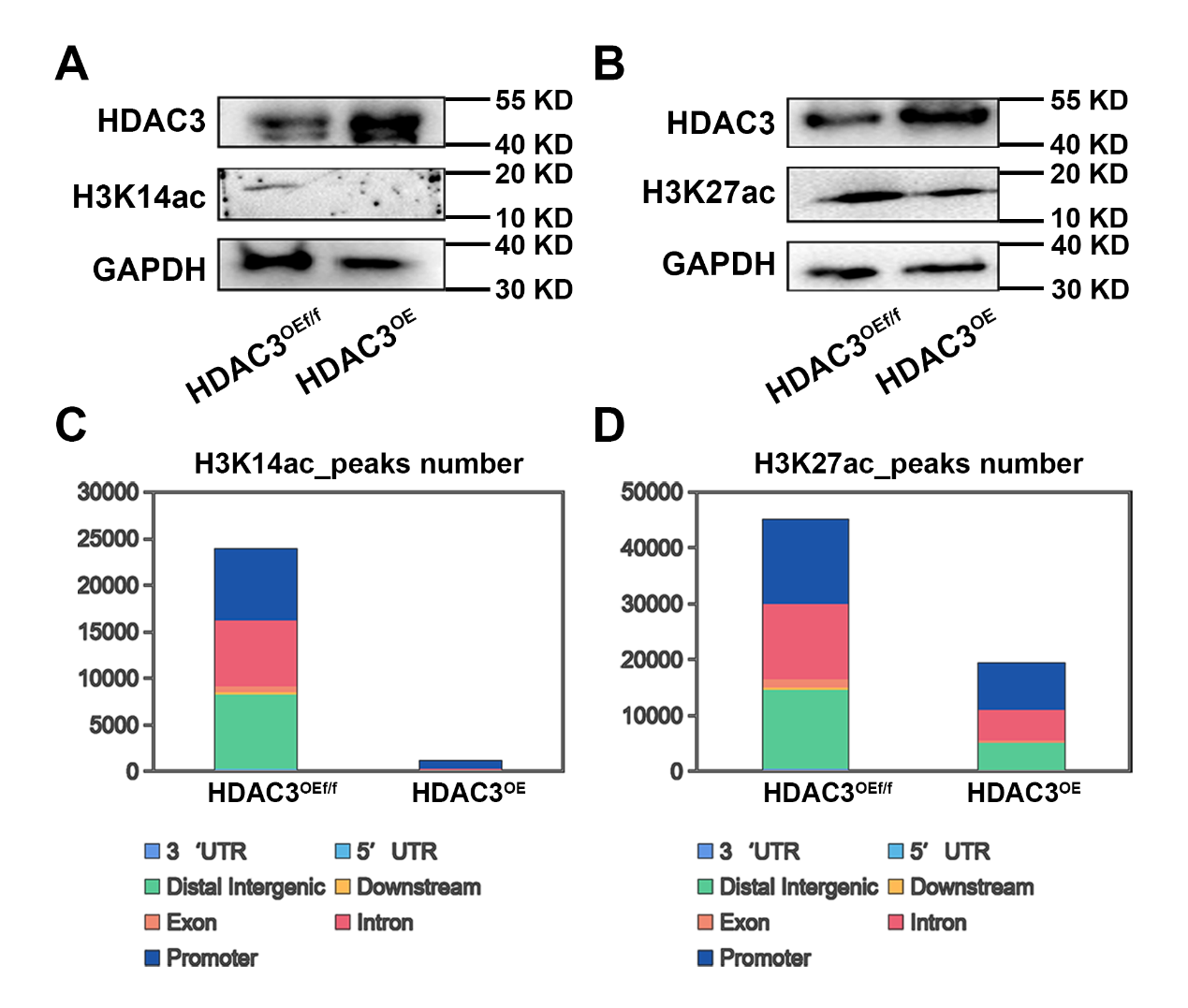


**Fig S4. HDAC3 repressed H3K14ac and H3K27ac levels in mice GCs.** (**A**) Immunoblotting analysis of H3K14ac in *Hdac3*^OE^ GCs and *Hdac3*^OEf/f^ GCs 4 hours after hCG induction. GAPDH was used as a loading control. (**B**) Immunoblotting analysis of H3K27ac in *Hdac3*^OE^ GCs and *Hdac3*^OEf/f^ GCs 4 hours after hCG induction. GAPDH was used as a loading control. (**C**) Diagram representing the peaks number of H3K14ac on the chromatins in *Hdac3*^OE^ GCs and *Hdac3*^OEf/f^ GCs 4 hours after hCG induction. (**D**) Diagram representing the peaks number of H3K27ac on the chromatins in *Hdac3*^OE^ GCs and *Hdac3*^OEf/f^ GCs 4 hours after hCG induction.


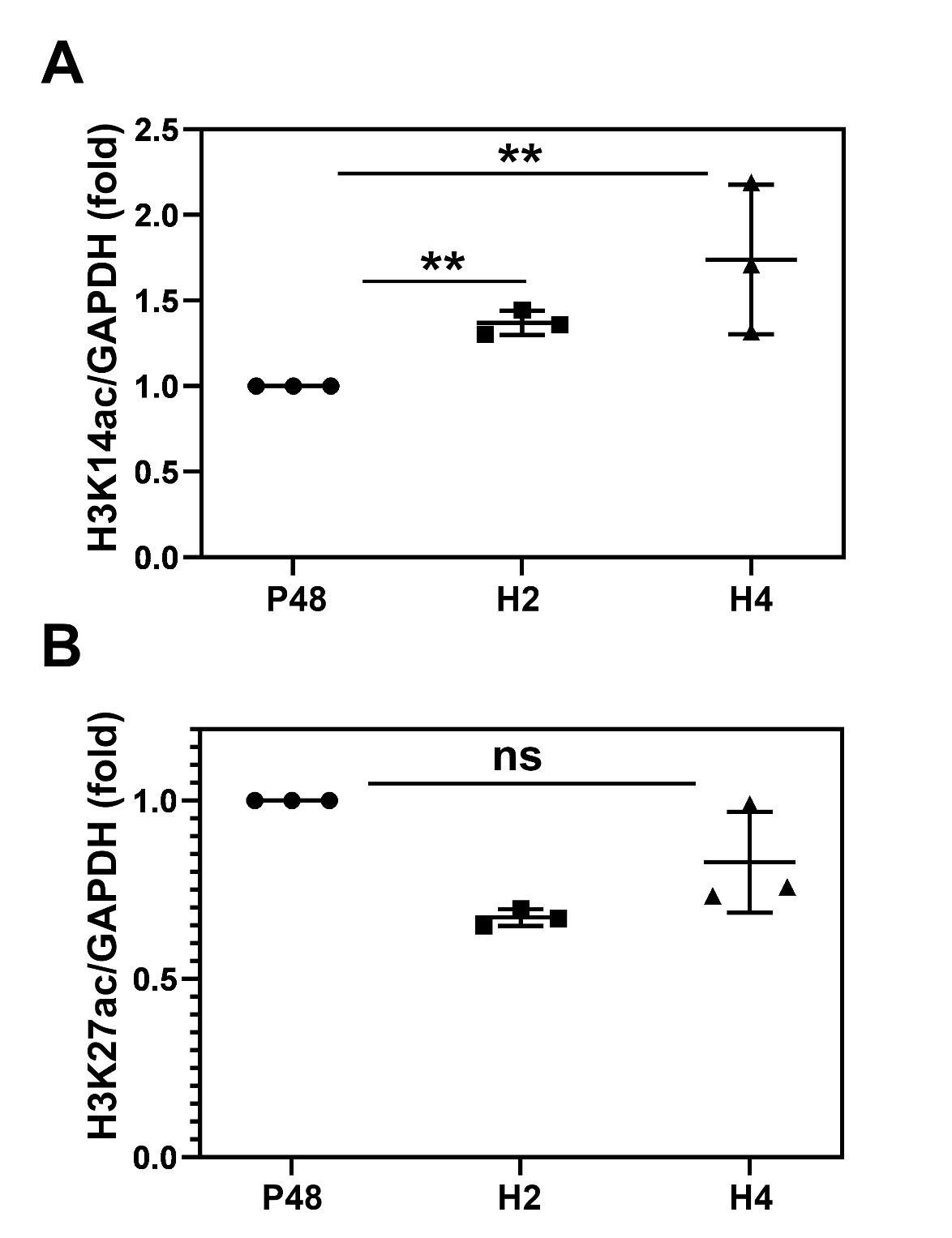


**Fig S5. Scatter graphs showed the gray-scale scans of H3K14ac and H3K27ac relative to GAPDH.** (**A**) Relative expression of H3K14ac in P48, H2, and H4 groups relative to GAPDH. (**B**) Relative expression of H3K27ac in P48, H2, and H4 groups relative to GAPDH. Graphs show the means ± SD of three independent experiments (n=3). *P*-values were performed using ANOVA, ***P* < 0.01; ns = not significant.


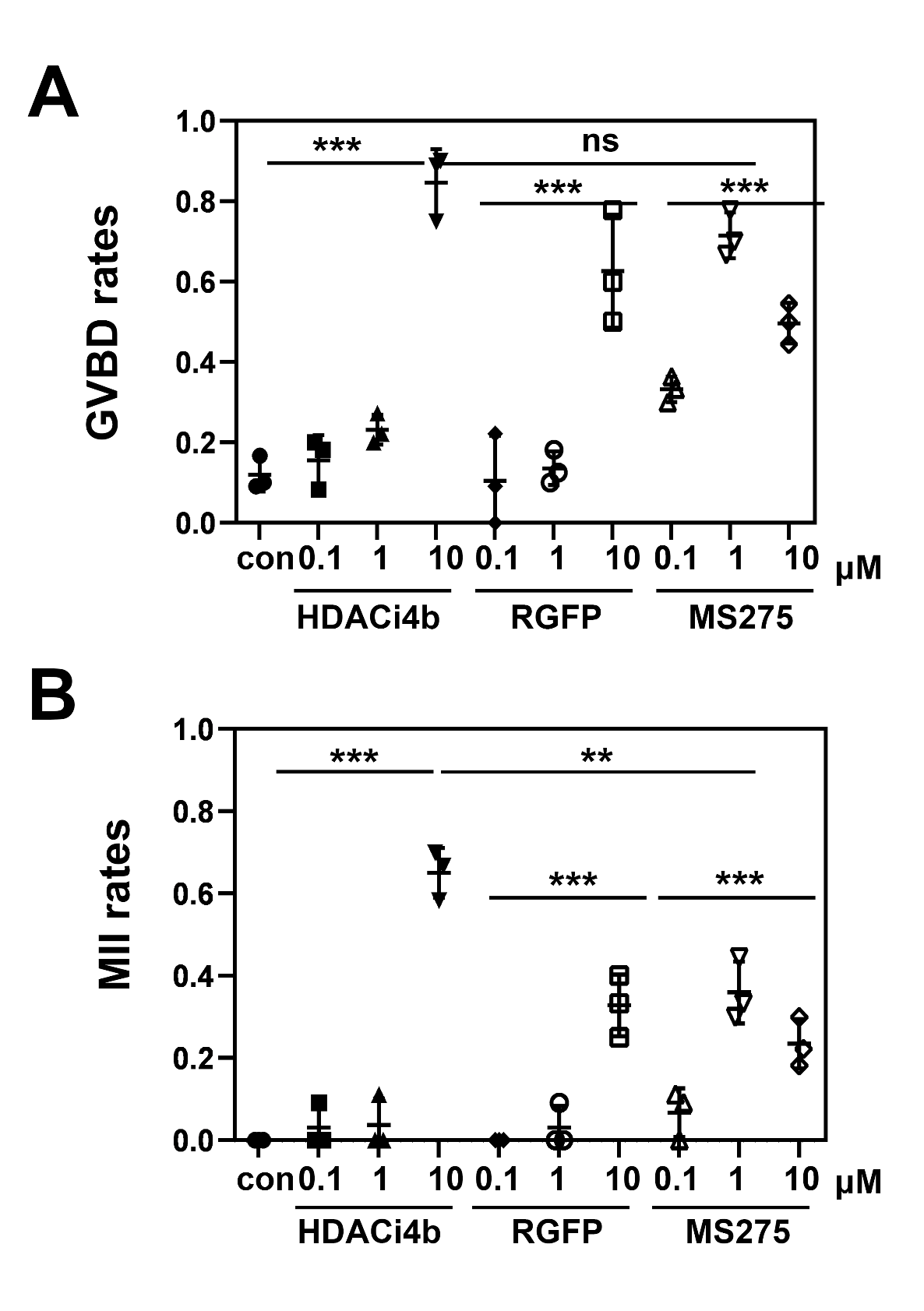


**Fig S6. The effects of three different HDAC3 inhibitors on IVM induction of oocytes in mouse preovulatory follicles.** (**A**) GVBD rates and (**B**) MII rates. Data are presented as means ± SD from three independent experiments (n=3). Statistical analysis was performed using ANOVA, with significance indicated as ***P* < 0.01, ****P* < 0.001; ns = not significant.

**Table S1**

| **Antibody** | **Company** | **Catalog** | **Application** |
| --- | --- | --- | --- |
| HDAC3 | Abcam | Ab7030 | IF, WB, Cut &Tag |
| EGFP | Abcam | ab184601 | IF, WB |
| FOXL2 | Novus | NB100-1277 | IF |
| Ki67 | CST | 9449 | IF |
| H3K14ac | Gene Text | GTX88008 | IF, WB, Cut &Tag |
| H3K27ac | Active Motif | 39685 | IF, WB, Cut &Tag |
| CYP11A1 | Abcam | Ab272494 | IHC |
| GAPDH | Abcam | Ab8245 | WB |

**Table S1. The information of antibodies used in the experiments.** IF: Immunofluorescence, WB: Western Blotting, CUT & Tag: the cleavage under target & tagmentation, IHC: Immunohistochemistry.

**Table S2**

| **Gene** | **species** | **Forward** | **Reversed** |
| --- | --- | --- | --- |
| *Foxl2* | Mus musculus | AACACCGGAGAAACCAGACC | CGTAGAACGGGAACTTGGCT |
| *AREG* | Homo sapiens | TGTCGCTCTTGATACTCGGC | ATGGTTCACGCTTCCCAGAG |
| *MMP9* | Homo sapiens | CGACGTCTTCCAGTACCGAG | GGGCACTGCAGGATGTCATA |
| *USH1C* | Homo sapiens | TGGGAAGGTGGTCGTTTCTG | GCCAGGGTGTAGTCTGTCAC |
| *CEP112* | Homo sapiens | ACCATCCATCTGCCAAGGAAA | TCCTGTATCCCTCAGAGGCT |
| *SERPING1* | Homo sapiens | CCAGAAGTTTGGAGTCCGCT | TGTTGTTGCGACCTTCCCTT |
| *FN11* | Homo sapiens | CCGCCGAATGTAGGACAAGA | TGTCAGAGTGGCACTGGTAG |
| *TENM4* | Homo sapiens | AAAGCCCCGCAGAAATCGTA | GAGGGGGCGTTACTTCTTCC |
| *AZGP1* | Homo sapiens | TGCAGGGAAGGTTTGGTTGT | TTGGTTATCTGGGCTGCTGG |

**Table S2. Sequence of primers used for quantitative PCR.**
