# Supplementary material for: Pathogenic mechanism of abnormal expression of HDAC3 in ovulatory granulosa cells inducing oocyte maturation disorder and its application in IVM
Source: J Biol Chem. 2025 Feb 10;301(3):108287. doi: 10.1016/j.jbc.2025.108287 (PMC11923827; doi:10.1016/j.jbc.2025.108287)

Figure S1C

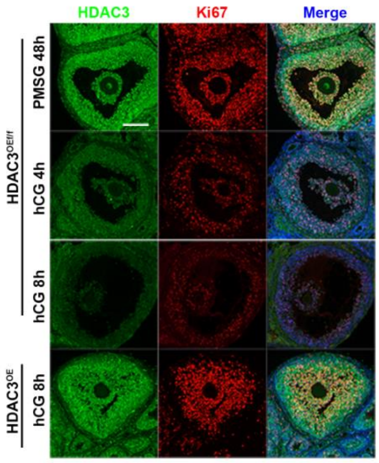

HDAC3^{OEff} PMSG 48h

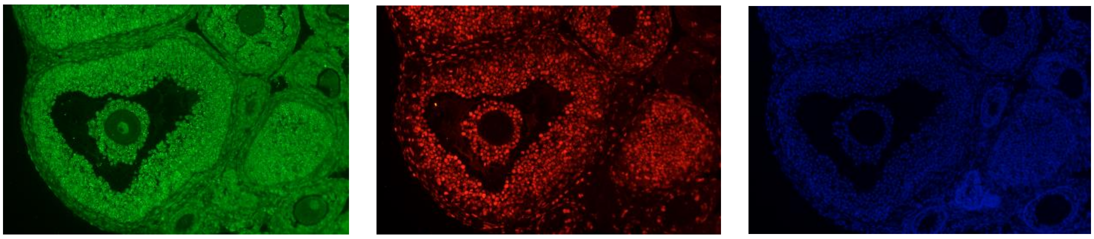

HDAC3^{OEff} hCG 4h

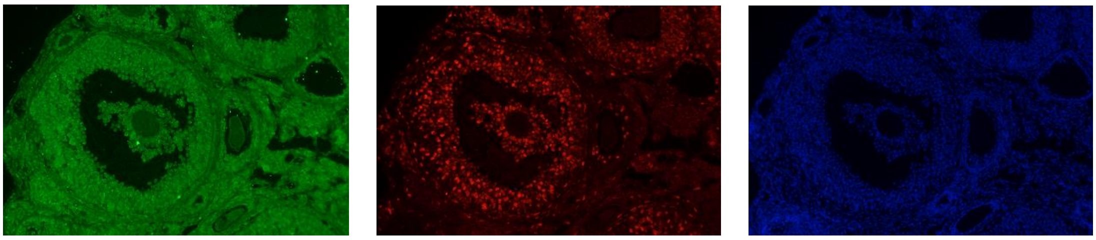

HDAC3^{OEff} hCG 8h

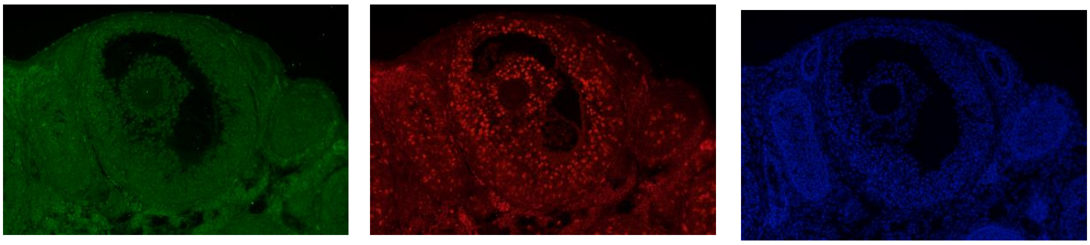

HDAC3^{OE} hCG 8h

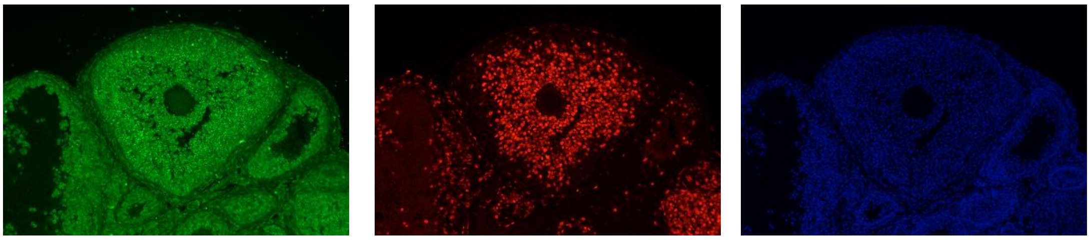

Figure 1B

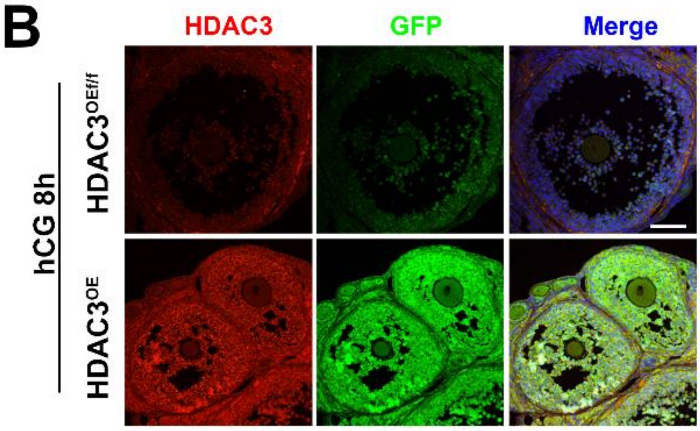

HDAC3$^{OEf/f}$

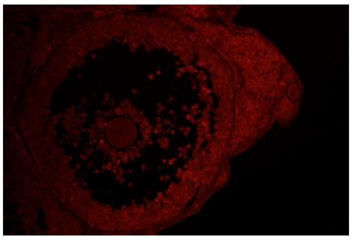

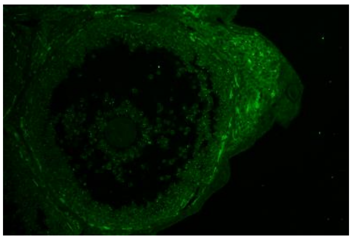

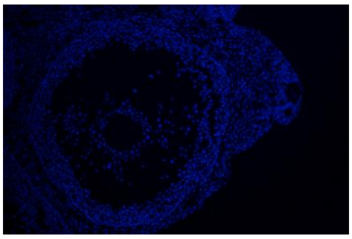

HDAC3^{OE}

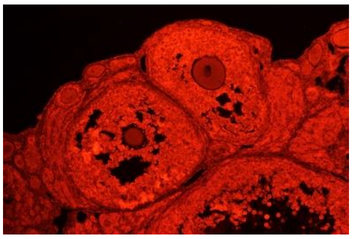

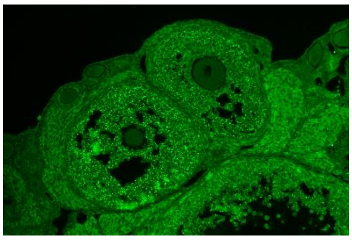

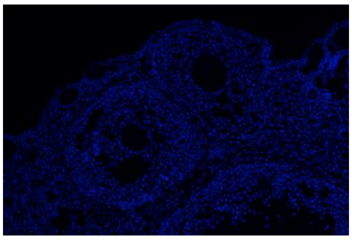

Figure 1H

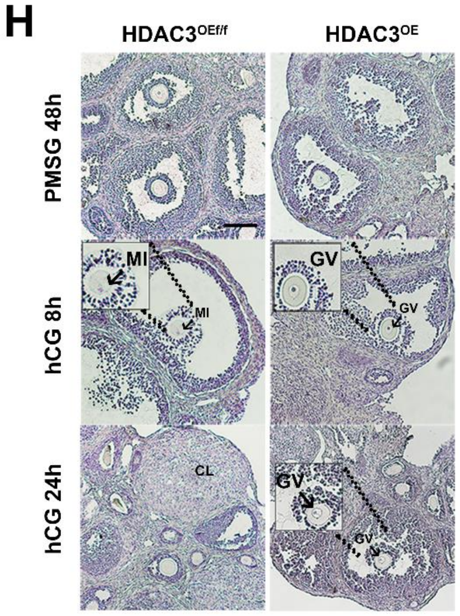

HDAC3$^{OEf/f}$

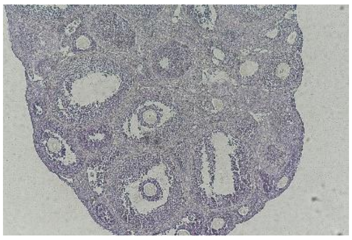

PMSG 48h

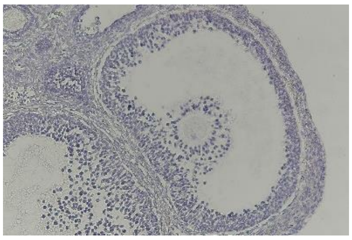

hCG 8h

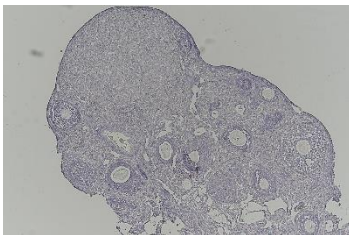

hCG 24h

HDAC3^{OE}

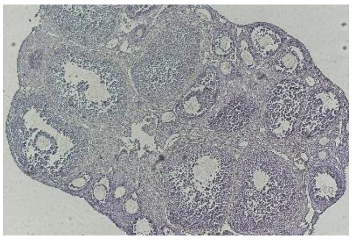

PMSG 48h

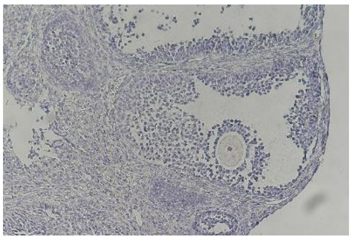

hCG 8h

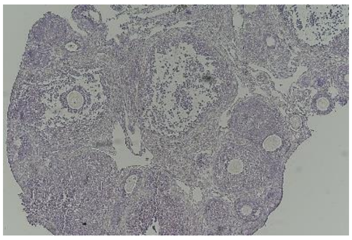

hCG 24h

Figure 2B

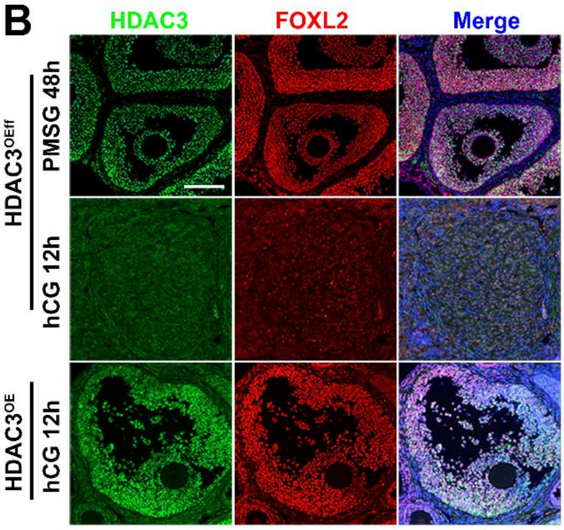

$HDAC3^{OEf/f}$
PMSG 48h

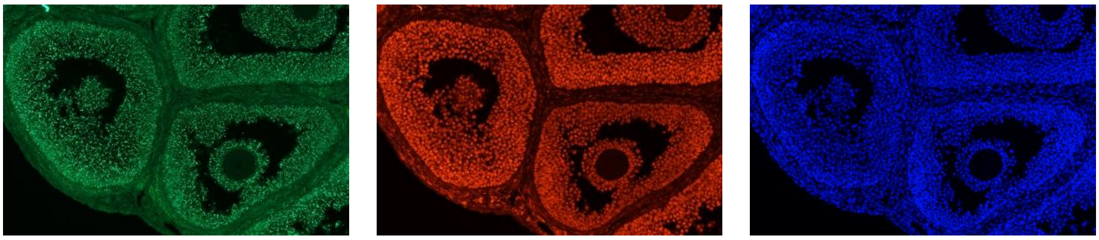

$HDAC3^{OEf/f}$
hCG 12h

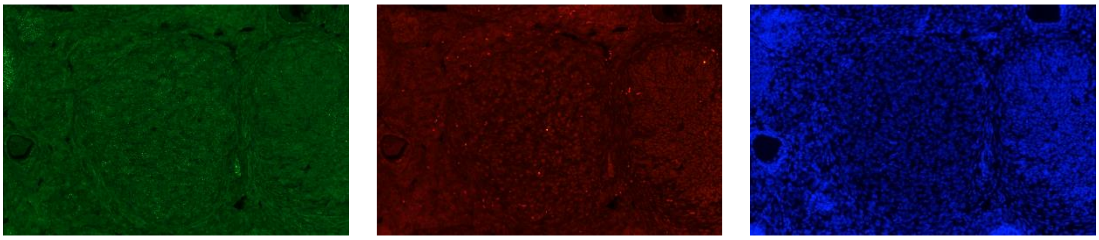

$HDAC3^{OE}$
hCG 12h

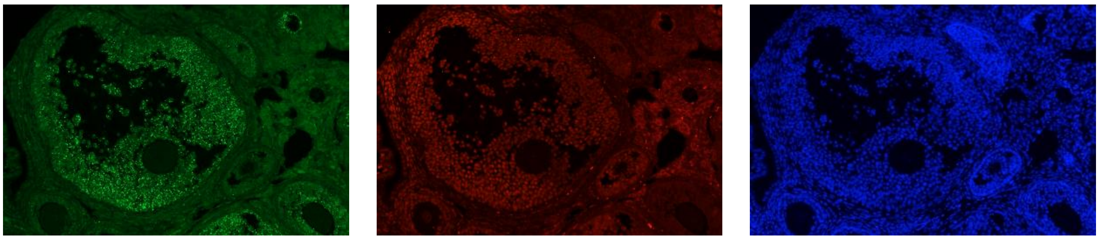

Figure 2D

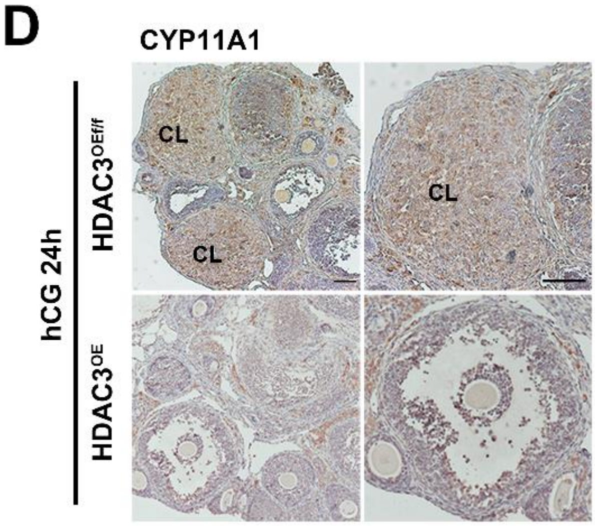

HDAC3$^{OEf/f}$

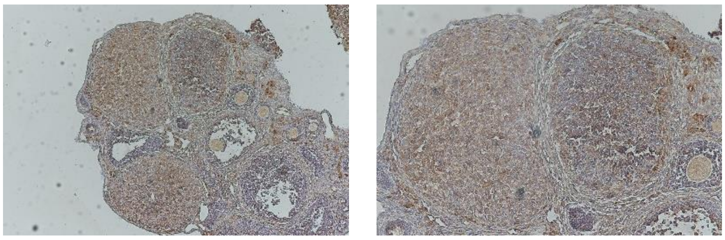

HDAC3^{OE}

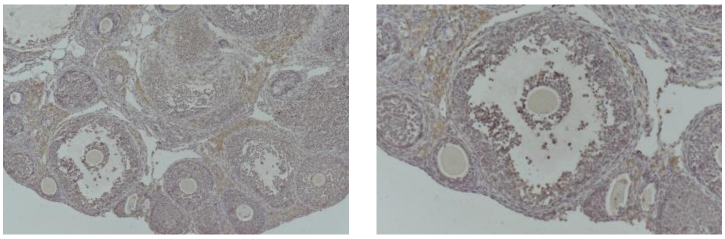

Figure 4A

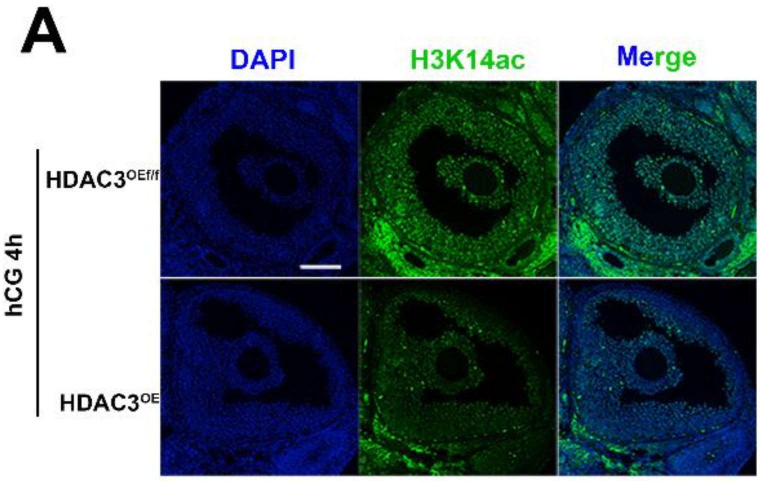

HDAC3$^{OEf/f}$

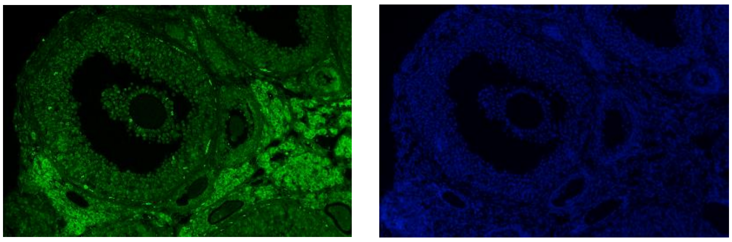

HDAC3^{OE}

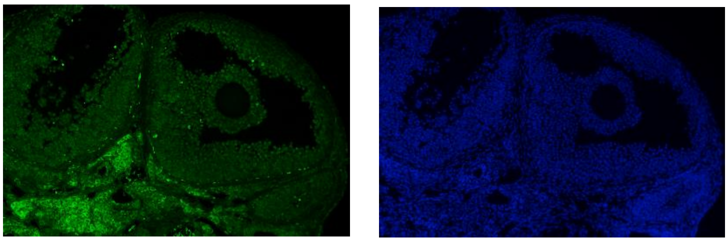

Figure 4B

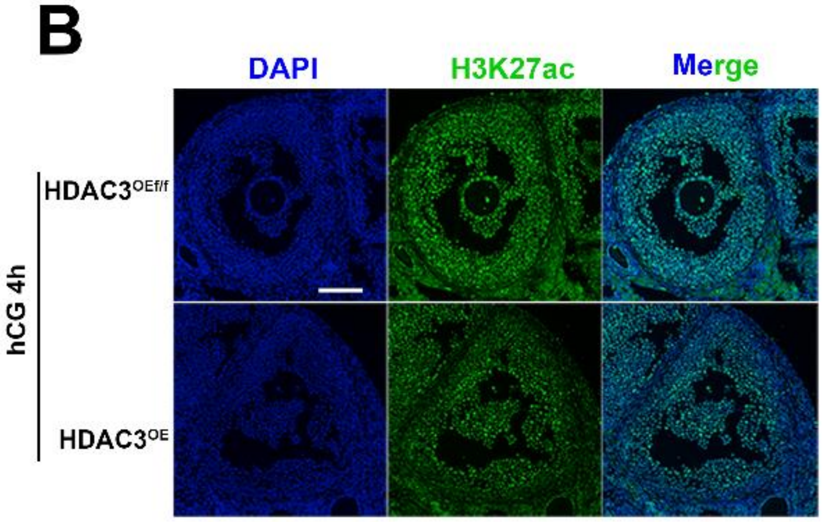

HDAC3$^{OEf/f}$

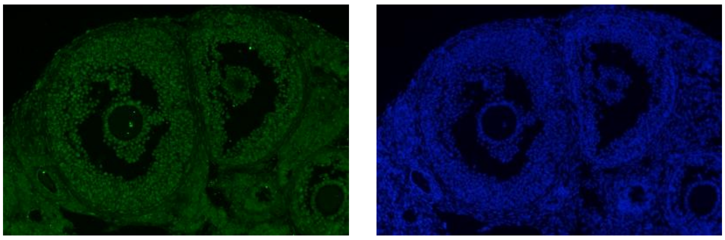

HDAC3^{OE}

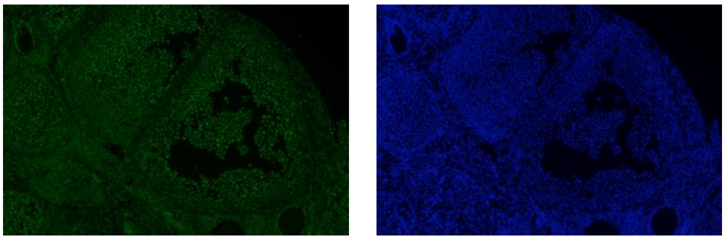

Western Blotting

Figure 1D

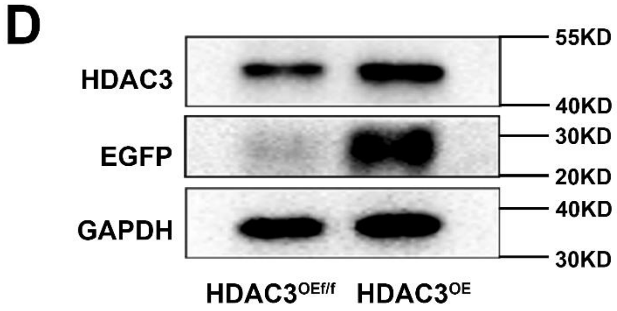

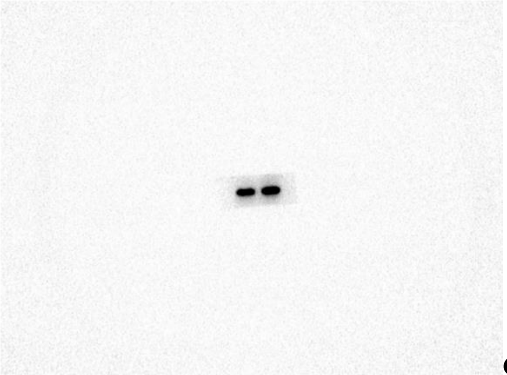

GAPDH（HDAC3^{OEff} vs HDAC3^{OE}）

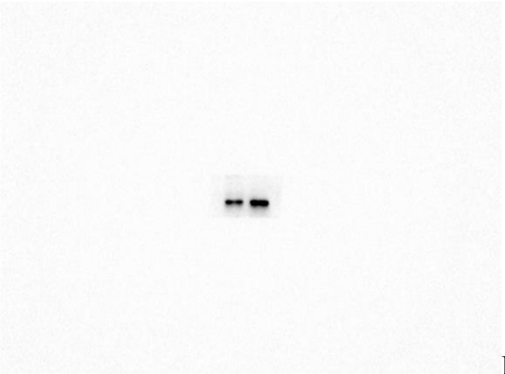

HDAC3（HDAC3^{OEff} vs HDAC3^{OE}）

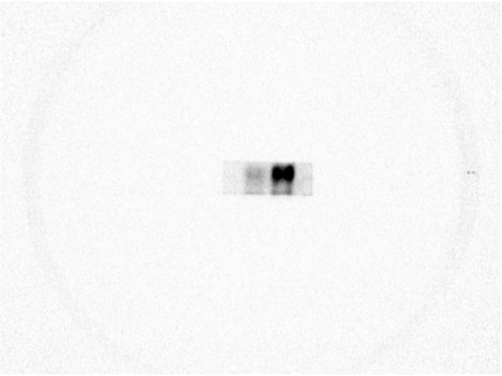

EGFP（HDAC3^{OEff} vs HDAC3^{OE}）

Figure 3D

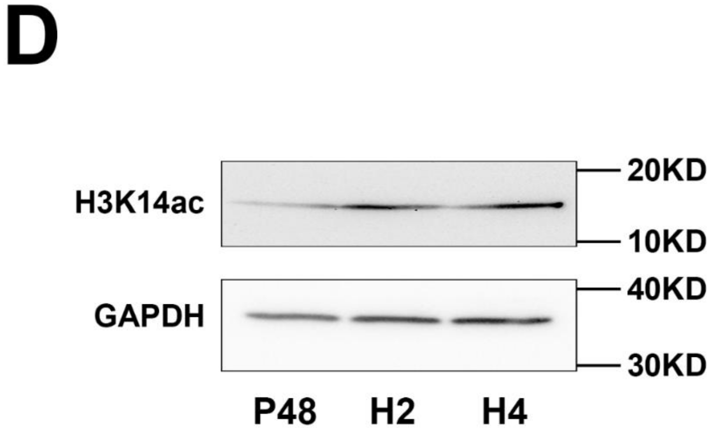

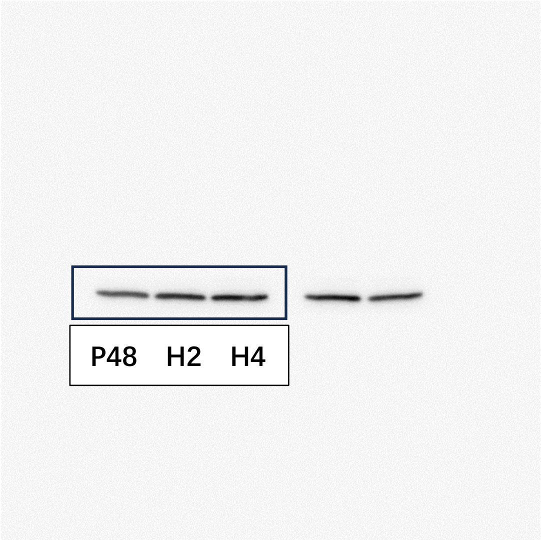

GAPDH （P48-H2-H4）

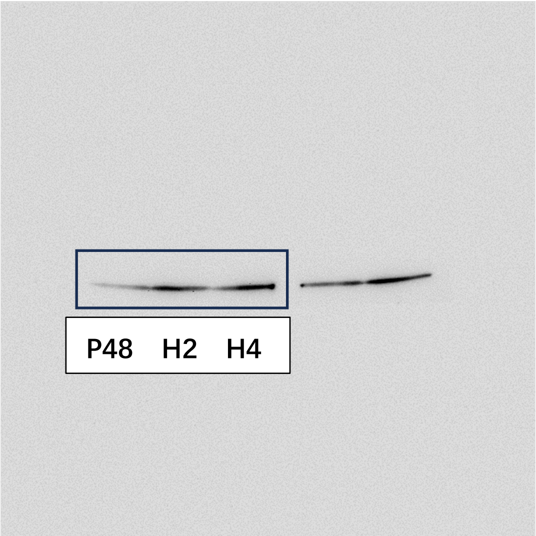

H3K14ac （P48-H2-H4）

Figure 3G

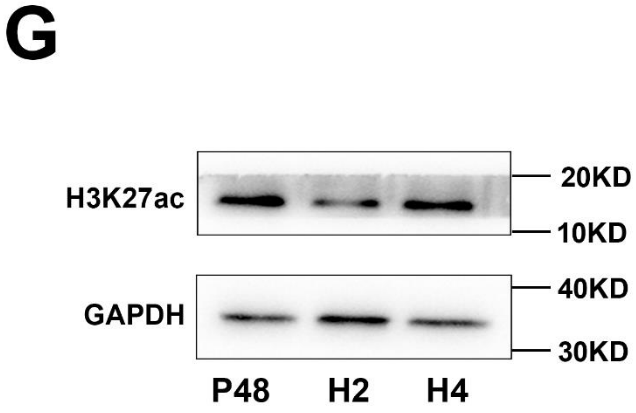

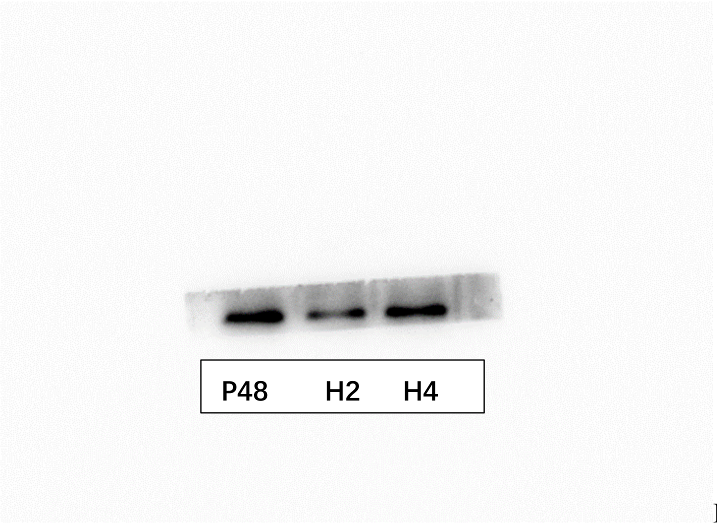

H3K27ac （P48-H2-H4）

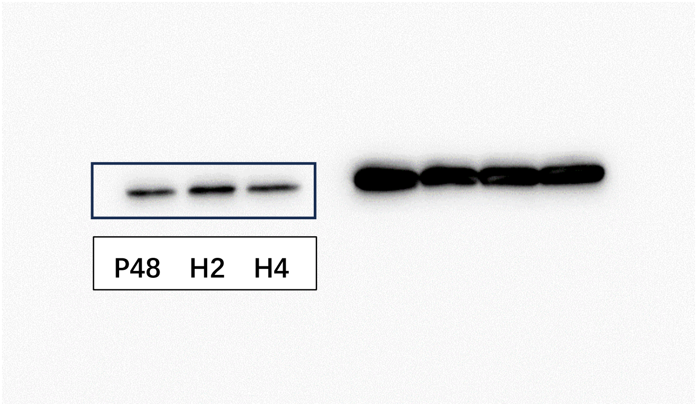

GAPDH （P48-H2-H4）

Figure 5D

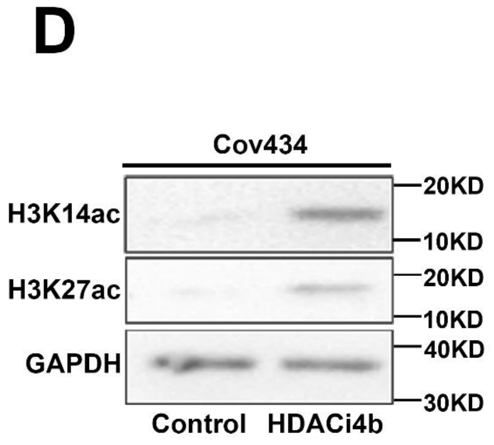

GAPDH （Control vs HDACi 4b）

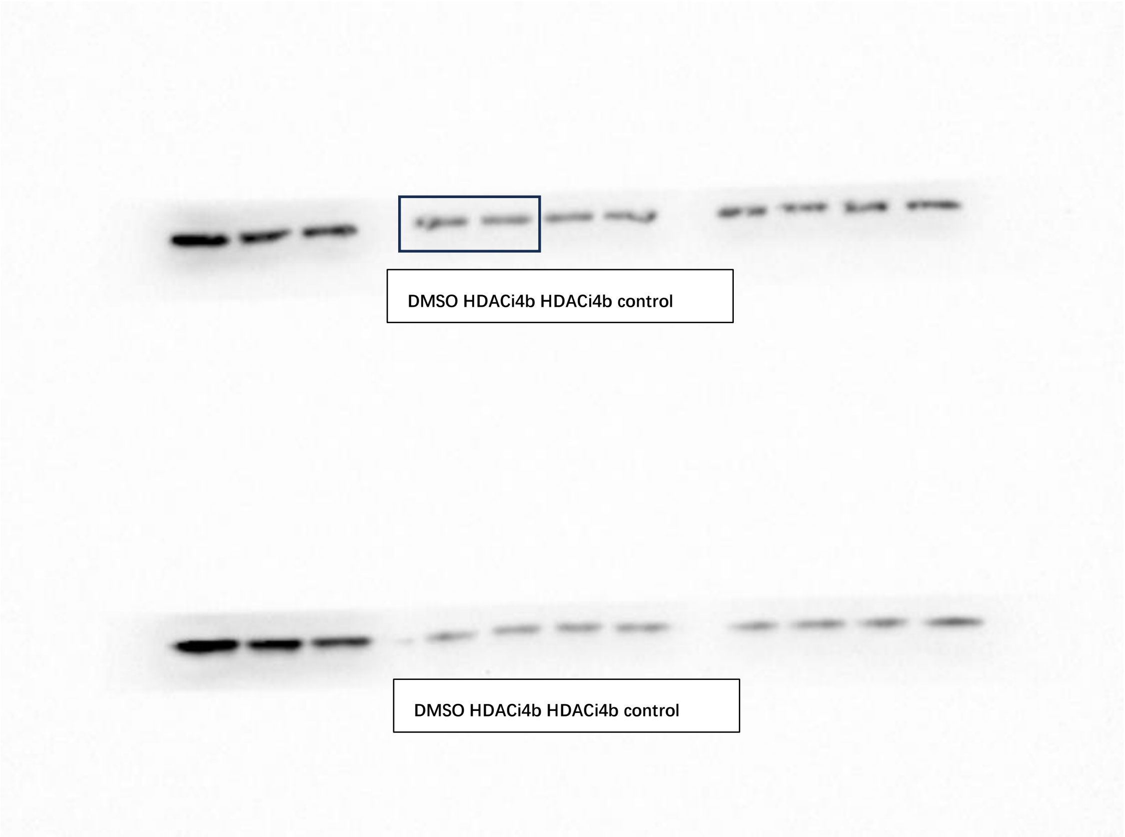

H3K14ac and H3K27ac （Control vs HDACi 4b）

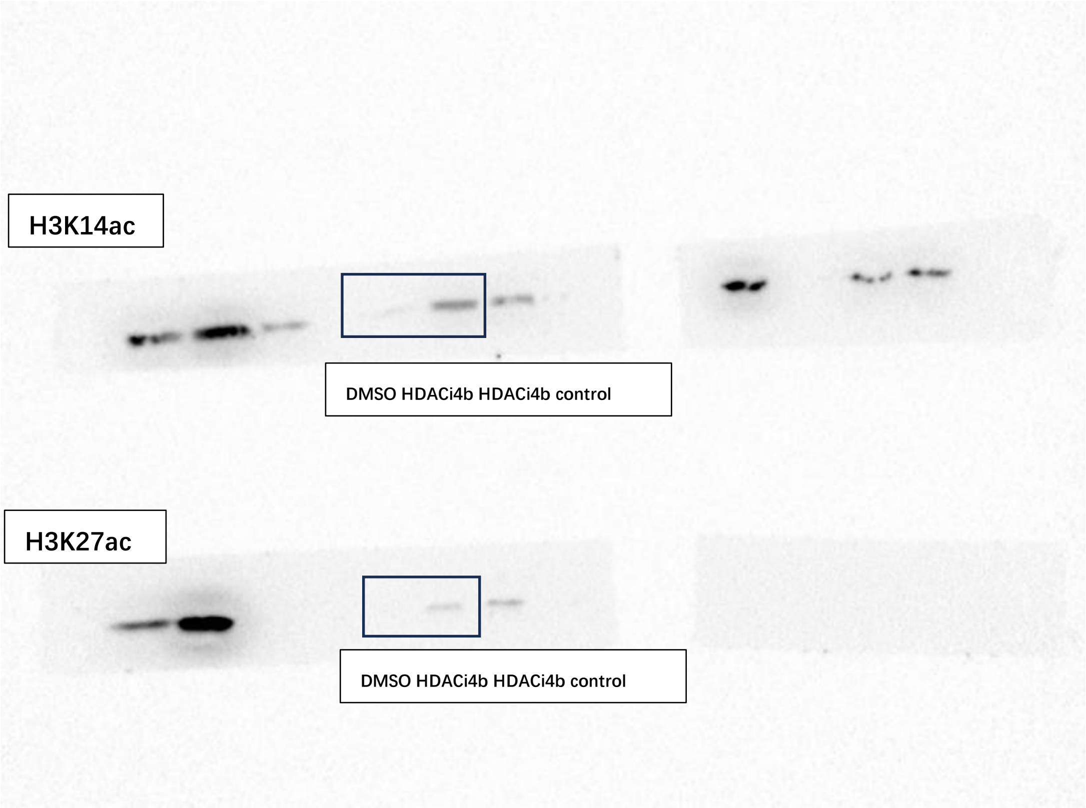

Supplement: Rawdata-1.6 [file mmc2.pdf]
